# Bariatric surgery may alleviate MASLD by restoring mitochondria-lipid droplet structural and functional integrity through suppression of the Mitochondrial Fission Factor-Perilipin 5 axis

**DOI:** 10.1186/s43556-026-00607-0

**Published:** 2026-10-06

**Authors:** Yuze Sun, Yuxuan Liu, Xiyu Chen, Shizhou Deng, Ning Zhang, Feifei Wu, Dong Fan, Yayun Wang, Yanling Yang

**Affiliations:** 1 Department of Hepatobiliary Surgery, Xi-Jing Hospital, Air Force Medical University, Xi’an, China; 2 Department of General Surgery, Hepatobiliary Surgery Center, Tangdu Hospital, The Fourth Military Medical University, Xi’an, 710038 China; 3 National Demonstration Center for Experimental Preclinical Medicine Education, Air Force Medical University, Xi’an, China; 4 Western Theater Command Air Force Hospital, Chengdu, China

**Keywords:** MASLD, Bariatric surgery, MFF, PLIN5, Mitochondria-LD contact

## Abstract

Metabolic dysfunction-associated steatotic liver disease (MASLD) is one of the most prevalent chronic liver diseases worldwide, characterized primarily by excessive lipid droplet (LD) accumulation in hepatocytes. Bariatric surgery, particularly sleeve gastrectomy (SG), represents the most effective intervention for reversing MASLD, although the underlying mechanisms remain incompletely understood. This study aimed to investigate the mitochondrial and lipid droplets (LDs) related molecular mechanisms by which SG ameliorated MASLD. Using a high-fat diet (HFD)-induced mouse model of MASLD, combined with SG intervention, hepatocyte-specific genetic manipulation, multi-omics analysis, and mitochondrial ultrastructural examination, we observed that MASLD was associated with a marked upregulation of perilipin 5 (PLIN5) on the LD surface and mitochondrial fission factor (MFF) on the mitochondrial outer membrane. Ultrastructural and co-immunoprecipitation (Co-IP) analyses revealed that PLIN5 and MFF formed a complex that facilitated the recruitment of LDs to mitochondria and their penetration into the mitochondrial matrix. This process ultimately led to disrupted mitochondrial fatty acid β-oxidation and structural disorganization. SG significantly downregulated PLIN5 and MFF expression, reduced LD-induced mitochondrial damage, and enhanced mitochondrial resilience to high-fat stress. These changes might contribute to the restoration of mitochondria-LD structural and functional homeostasis, ultimately reversing MASLD. Our findings indicated that aberrant upregulation of MFF contributed to MASLD progression, and SG might alleviate the disease by suppressing the MFF-PLIN5 axis to normalize mitochondria-LD interactions. These results suggested that targeting the MFF-PLIN5 axis may offer a promising therapeutic strategy for non-surgical management of MASLD.

**Supplementary Information:**

The online version contains supplementary material available at https://doi.org/10.1186/s43556-026-00607-0.

## Introduction

Metabolic dysfunction-associated steatotic liver disease (MASLD) encompasses a spectrum from simple hepatic steatosis (metabolic dysfunction-associated steatotic liver, MASL) to metabolic dysfunction-associated steatohepatitis (MASH), which can progress to fibrosis, cirrhosis, and hepatocellular carcinoma [1]. Concurrently, MASLD is closely associated with obesity, insulin resistance, and type 2 diabetes and is also a major risk factor for cardiovascular disease, kidney disease, and extrahepatic malignancies. Affecting approximately 35% of adults worldwide, MASLD represents a substantial public health burden [2, 3].

Therapeutic options for MASLD remain limited. Lifestyle intervention is the cornerstone of management, but poor long-term adherence often limits sustained weight loss [4]. Bariatric surgery, particularly sleeve gastrectomy (SG), is among the most effective approaches for reversing MASLD [5, 6], producing durable weight loss and marked improvements in hepatic steatosis, inflammation, and fibrosis [7, 8]. Its metabolic benefits extend beyond weight reduction and involve changes in gastrointestinal hormones, bile acid metabolism, and the gut microbiota [9–12]. Enhanced hepatic mitochondrial oxidative phosphorylation (OXPHOS) after surgery further suggests weight loss-independent effects on hepatocellular metabolism [13].

Although lipid droplet (LD) accumulation has traditionally been considered a central driver of MASLD [14], increasing evidence indicates that mitochondrial dysfunction may also have a primary role because mitochondria are essential for fatty acid β-oxidation and energy production [15, 16]. The “multiple-hit” hypothesis further highlights oxidative stress and mitochondrial injury as important contributors to MASH progression [17].

Recent attention has therefore shifted toward interorganellar communication, particularly mitochondria-LD contact sites that regulate fatty acid transfer and oxidation [18]. Perilipin 5 (PLIN5), an LD-associated protein enriched in oxidative tissues, promotes mitochondrial recruitment to LDs and fatty acid utilization [19, 20]. Mitochondrial fission factor (MFF), a key regulator of mitochondrial division, is associated with mitochondrial fragmentation and dysfunction in metabolic diseases [21, 22]. However, its role in mitochondria-LD interactions during MASLD remains unclear.

Here, using high-fat diet (HFD)-induced MASLD mouse model with SG intervention, bioinformatic analysis, liver-specific gene modulation, and ultrastructural examination, we investigated how SG regulates mitochondrial dynamics and mitochondria-LD interactions in hepatocytes. We identified aberrant MFF upregulation and its interaction with PLIN5 as a mechanism promoting pathological mitochondria-LD contact and mitochondrial dysfunction. SG suppressed the MFF-PLIN5 axis, restored physiological organelle interactions and mitochondrial function, and ameliorated hepatic steatosis and injury, suggesting the MFF-PLIN5 axis as a potential non-surgical therapeutic target for MASLD.

## Results

### HFD-induced MASLD in mice led to glucose and lipid metabolism dysregulation accompanied by mitochondrial dysfunction

**Fig. 1:**
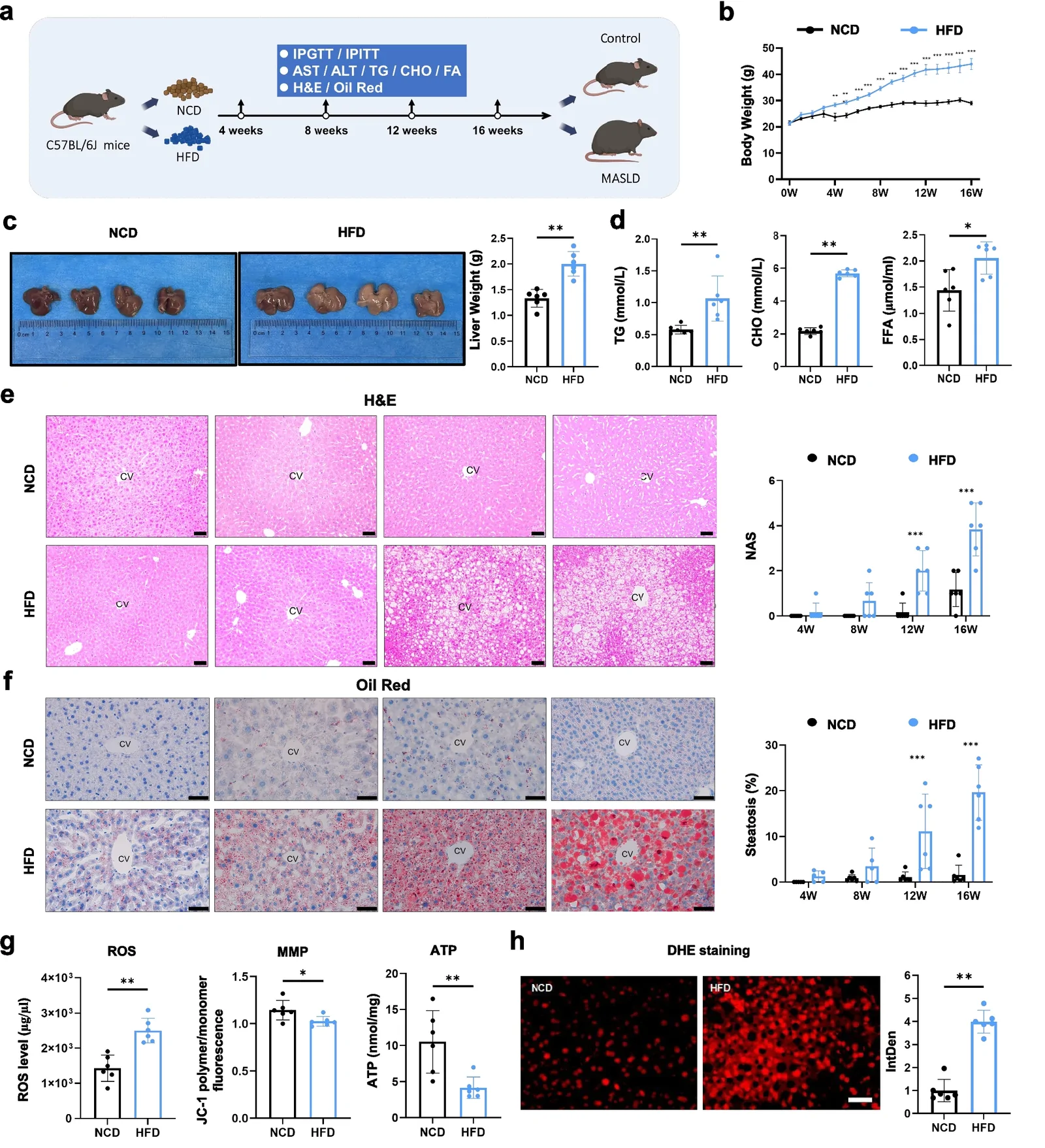
HFD-induced MASLD in mice led to glucose and lipid metabolism dysregulation accompanied by mitochondrial dysfunction. **a** Schematic of feeding HFD or NCD groups. **b** Gains of body weight along 4, 8, 12, and 16 weeks of HFD feeding. **c** Representative liver tissue images collected at 16 weeks after feeding, in NCD mice (left) and in HFD-fed mice (right). Quantitative results of livers weight. **d** ELISA results for serum TG, CHO and FFA at 16 weeks after feeding. **e** Liver H&E staining images (scale bar, 50 μm) at four time points (left) and the calculated NAS (right). CV represented the central vein. **f** Liver Oil Red O staining images (scale bar, 50 μm) at four time points (left) and the calculated steatosis percentage (right). CV represented the central vein. **g** Liver ROS levels, MMP and ATP content at 16 weeks after feeding. **h** DHE (ROS fluorescent probe) staining of liver tissue sections of NCD and HFD-fed mice (scale bar: 100 μm). Quantitative results of mean fluorescence intensity. *N* = 6 per group per time point in all assays. The statistical data for all panels were presented as the mean ± SD. ∗ *p* < 0.05, ∗∗ *p* < 0.01, ∗∗∗ *p* < 0.001

To explore the dynamic relationship between aberrant lipid metabolism and mitochondrial dysfunction during MASLD progression, we established a mouse model by feeding C57BL/6 J mice a HFD for 16 weeks (Fig. 1a). Compared with the normal chow diet (NCD) group, HFD-fed mice showed progressive weight gain from week 4 and a six-fold increase in fat mass, whereas lean mass remained unchanged (Fig. 1b and Fig. S1a). Their livers were enlarged and pale, and serum alanine aminotransferase (ALT), aspartate aminotransferase (AST), triglycerides (TG), cholesterol (CHO), and free fatty acids (FFA) levels progressively increased, indicating hepatic injury and dyslipidemia (Fig. 1c, d and Fig. S1b). HFD feeding also impaired glucose tolerance and insulin sensitivity from week 4 through week 16 (Fig. S1c, d). H&E staining showed increased lipid vacuoles and higher NAFLD activity scores (NAS) at weeks 12 and 16, and Oil Red O staining confirmed marked hepatic LD accumulation (Fig. 1e, f).

To further assess mitochondrial function alongside the observed metabolic alterations, we measured reactive oxygen species (ROS) levels, mitochondrial membrane potential (MMP), and ATP production. HFD feeding significantly increased ROS, decreased MMP, and reduced ATP production by approximately 50% (Fig. 1g). Dihydroethidium (DHE) staining further showed a four-fold increase in hepatic superoxide-associated fluorescence (Fig. 1h). Similar changes were observed in the oleic acid (OA) + palmitic acid (PA)-induced cellular model, including increased LD accumulation, reduced ATP production and MMP, ROS accumulation, and a 60% reduction in fatty acid oxidation (FAO) activity (Fig. S2a-e). Oxygen consumption rate (OCR) analysis confirmed impaired mitochondrial respiration (Fig. S2f). Fatty acid synthesis increased, whereas mitochondrial β-oxidation decreased in vitro (Fig. S2g, h). To validate these findings in vivo, we examined the expression of fatty acid metabolism genes in liver tissues from NCD and HFD-fed mice. Consistent with the cellular model, HFD feeding significantly upregulated fatty acid synthesis genes and downregulated β-oxidation genes compared to NCD controls (Fig. S3a, b), confirming that MASLD was associated with a reprogramming of hepatic lipid metabolism toward increased lipogenesis and reduced fatty acid oxidation. These results indicated that the pathological state of MASLD led to hepatic mitochondrial dysfunction.

### Bariatric surgery effectively ameliorated MASLD symptoms and reversed mitochondrial dysfunction

**Fig. 2:**
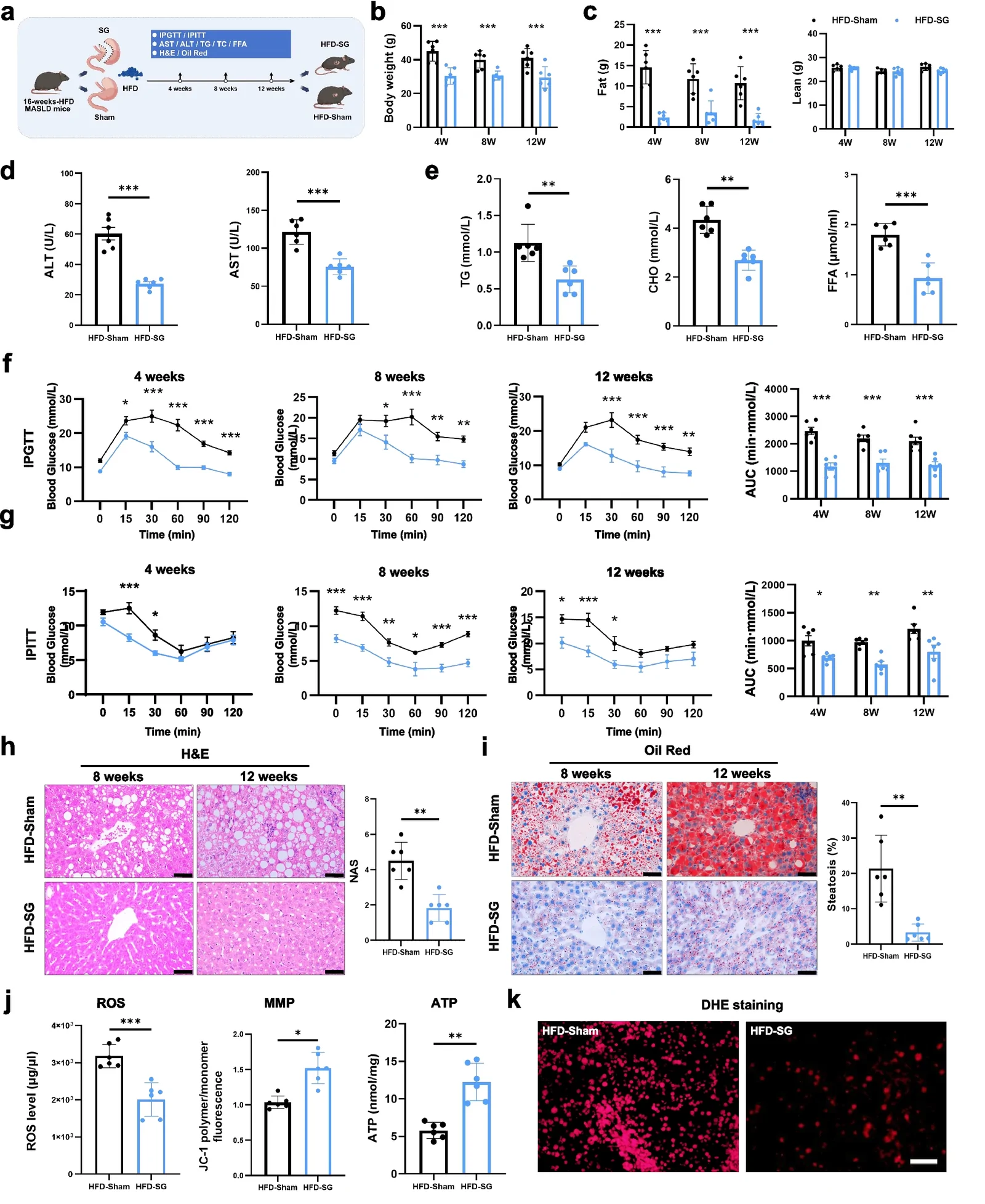
SG effectively alleviated the symptoms of MASLD and reversed mitochondrial dysfunction. **a** Schematic diagram of operation procedure. According to the operation scheme, HFD-fed mice were divided into HFD-Sham and HFD-SG groups. **b** Weight loss along 4, 8, and 12 weeks after surgery. **c** Fat content and lean content along 4, 8, and 12 weeks after surgery. **d** ELISA results for serum ALT and AST at 8 weeks after surgery. **e** ELISA results for serum TG, CHO and FFA at 8 weeks after surgery. **f** IPGTT and their calculated AUC at three time points. **g** IPITT and their calculated AUC at three time points. **h** Liver H&E staining (scale bar: 100 μm) at 8 and 12 weeks after surgery (left) and NAS scores (right). **i** Liver Oil Red O staining (scale bar: 100 μm) at 8 and 12 weeks after surgery (left) and steatosis percentage (right). **j** Liver ROS levels, MMP and ATP content at 8 weeks after surgery. **k** DHE staining of liver tissue sections at 8 weeks after surgery (scale bar: 100 μm). *N* = 6 per group per time point in all assays. The statistical data for all panels were presented as the mean ± SD. ∗ *p* < 0.05, ∗∗ *p* < 0.01, ∗∗∗ *p* < 0.001

To better simulate clinical conditions and evaluate the therapeutic effect of bariatric surgery on MASLD, we performed SG in mice (Fig. 2a). The surgical procedure was adapted from established protocols described by Karen Ryan [23] and Jae Hoon Shin [24]. Our results demonstrated that SG effectively reversed MASLD related parameters in HFD-fed mice, including body weight, lean-to-fat tissue ratio, liver injury markers, lipid accumulation, glucose metabolism disturbances, and intrahepatic LD deposition. Compared with sham surgery, SG produced significant weight loss from postoperative week 4 through week 12 and markedly reduced fat mass without affecting lean mass (Fig. 2b, c). At 8 weeks, serum ALT, AST, TG, CHO, and FFA levels were reduced by approximately 40%, and glucose tolerance and insulin sensitivity improved from postoperative week 4 onward (Fig. 2d-g). H&E and Oil Red O staining at weeks 8 and 12 showed reduced hepatic lipid vacuoles, lobular inflammation, hepatocyte ballooning, NAS, and LD accumulation (Fig. 2h, i), indicating sustained improvement in hepatic steatosis. Together, these findings indicated that SG alleviated hepatic steatosis in MASLD mice, with effects maintained for at least 12 weeks. At the mitochondrial level, SG also demonstrated robust restorative effects. To directly assess mitochondrial function, we isolated mitochondria from liver tissues and measured ROS levels, MMP, and ATP production. SG also reduced mitochondrial ROS, restored MMP to near-normal levels, and approximately doubled ATP production (Fig. 2j). In parallel, DHE staining confirmed reduced hepatic oxidative stress (Fig. 2k). SG reversed HFD-induced fatty acid metabolic changes by downregulating fatty acid synthesis genes and upregulating β-oxidation genes (Fig. S4a, b). To distinguish these effects from caloric restriction (CR), a Sham + CR group was pair-fed to match SG caloric intake (Fig. S5a). SG produced greater improvements than Sham + CR in hepatic steatosis, ATP production, MMP, and ROS levels (Fig. S5b-g) and more strongly downregulated MFF and PLIN5 protein expression (Fig. S5h), indicating that SG exerted weight-independent effects on the MFF-PLIN5 axis. These data suggested that bariatric surgery not only reversed the macroscopic metabolic phenotype of MASLD but also restored mitochondrial function at the organelle level.

The significant alleviation of MASLD and related mitochondrial dysfunction by SG suggested that its mechanism of action may go beyond simple weight reduction and caloric restriction, instead involving a deeper molecular regulatory network. However, the specific molecular targets through which the surgery remodels hepatocyte metabolic homeostasis, particularly mitochondrial function repair, remain a critical unanswered scientific question.

### Bioinformatics analysis identified MFF as a key molecule linking MASLD to mitochondrial dysfunction

**Fig. 3:**
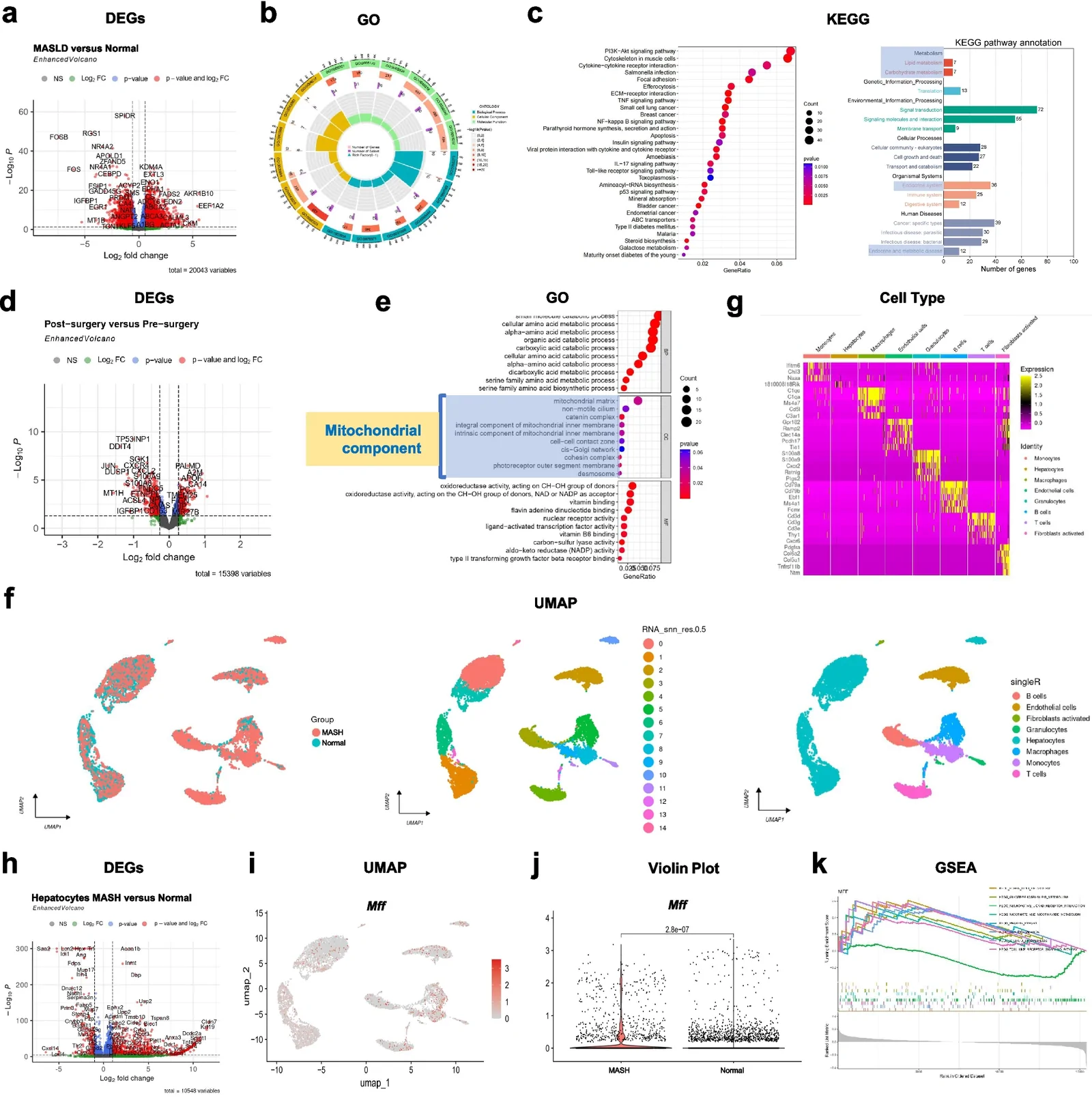
Multimodal bioinformatics analysis identified *Mff* as the key molecule linking MASLD and mitochondrial function. **a** Volcano plots of DEGs between healthy and MASLD patients, with the threshold of *P* < 0.05 and |log FC|> 0.5. **b** GO and **c** KEGG enrichment analysis of the DEGs. **d** Volcano plots of DEGs before and after metabolic bariatric surgery. **e** GO enrichment analysis of the DEGs. **f** GSE129516 collected single-cell sequencing data from liver tissues of MASH and normal mice. After cell clustering, cell annotation was performed in our Single R package. **g** The heat map showed the expression of marker genes in each cell group. **h** DEGs in Hepatocytes of MASH and healthy groups. **i** UMAP showed the expression ranges and levels of *Mff*. **j** The violin diagram showed the difference in expression levels of *Mff* between MASH-Hepatocytes and Normal-Hepatocytes. **k** GSEA of *Mff* in HALLMARK gene set

To identify the key molecular hubs linking MASLD to mitochondrial dysfunction and surgical efficacy, we systematically integrated transcriptomic data from clinical cohorts and preclinical models to screen for core regulatory factors associated with MASLD severity, surgical efficacy, and mitochondrial function. In GSE135251, comparison of MASLD and healthy liver tissues identified 2,127 differentially expressed genes (DEGs) (Fig. 3a). Gene Ontology (GO) analysis showed enrichment in adipocyte differentiation, ERK signaling, collagen-containing extracellular matrix, basement membrane, extracellular matrix structural constituents, and glycosaminoglycan binding, while KEGG analysis highlighted lipid and carbohydrate metabolism pathways (Fig. 3b, c), which aligned closely with the core metabolic disturbances observed in our HFD model.

Our next step was to investigate the molecular basis of bariatric surgery’s therapeutic effects. In GSE106737, analysis of liver samples before and after bariatric surgery identified 629 DEGs (Fig. 3d). In addition to lipid metabolism, GO enrichment included mitochondrial matrix and mitochondrial inner membrane components (Fig. 3e). Single-cell RNA sequencing data from GSE129516 were then analyzed to resolve cell-specific changes in MASH and normal mouse livers (Fig. 3f, g). Hepatocytes showed 1,161 upregulated and 220 downregulated genes in MASH (Fig. 3h). *MFF* was most highly expressed in hepatocytes and was significantly upregulated in MASH hepatocytes (Fig. 3i, j). Gene set enrichment analysis (GSEA) further showed a positive association between *MFF* expression and lipid metabolism pathways (Fig. 3k).

In summary, these analyses identified *MFF* as a pivotal candidate gene markedly upregulated in MASLD and strongly correlated with lipid metabolism dysregulation.

### SG effectively reversed HFD-induced upregulation of MFF expression and ameliorated mitochondrial ultrastructural damage and electron transport chain (ETC) impairment in the MASLD mice model

**Fig. 4:**
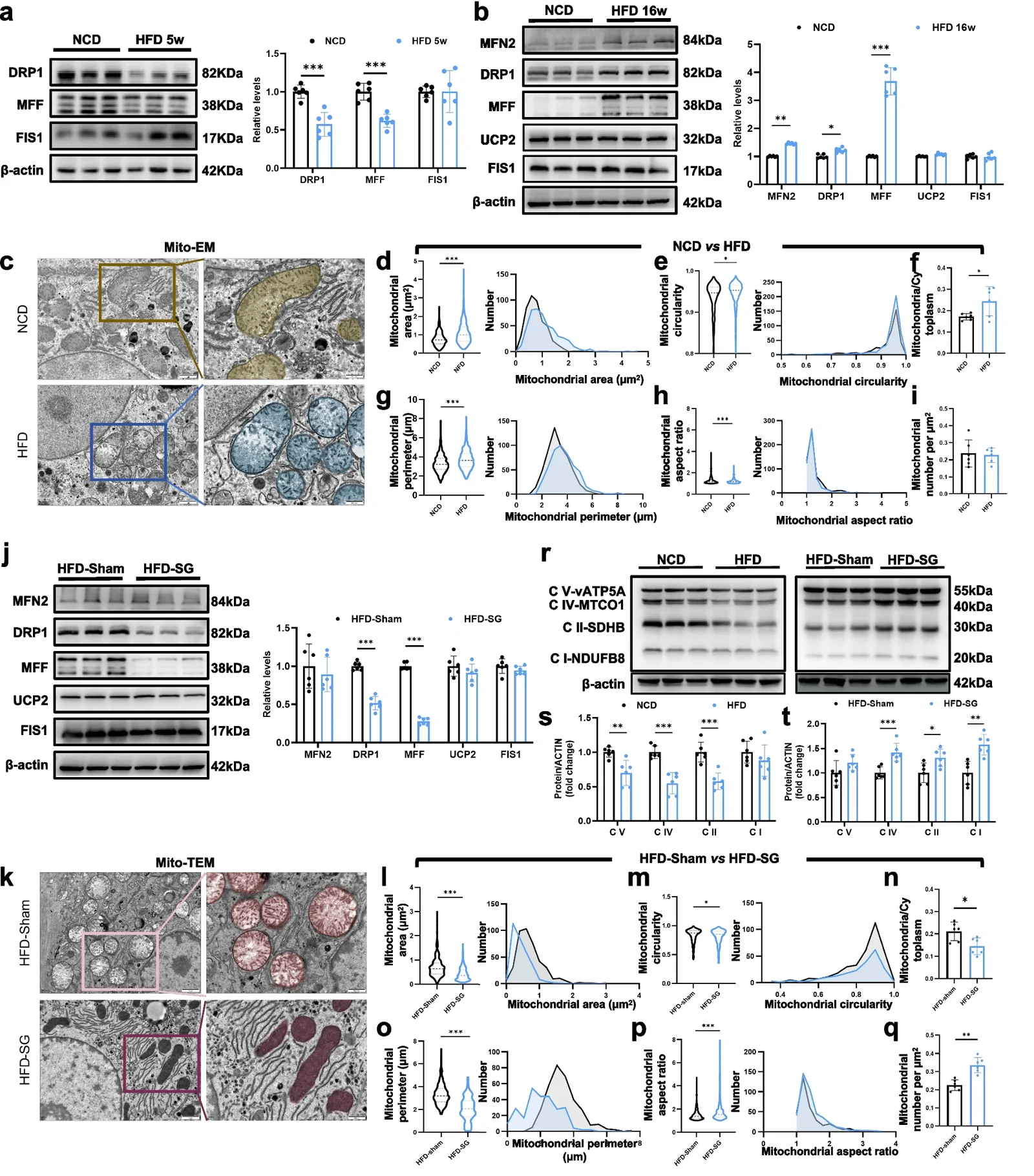
The upregulation of MFF expression in the MASLD model damaged mitochondrial membrane structure and the ETC, which was reversed by SG. **a** Protein expression related to liver mitochondrial fission at 5 weeks of feeding (left); quantitative results of mitochondrial fission related proteins (right). **b** Protein expression related to liver mitochondrial fission and fusion at 16 weeks of feeding (left); quantitative results of mitochondrial fission and fusion related proteins (right). **c** TEM results of liver mitochondria in NCD and HFD groups. **d-i** Mitochondrial area, mitochondrial circularity, ratio of mitochondrial area to cytoplasmic area, mitochondrial perimeter, mitochondrial aspect ratio, number of mitochondria per unit area of liver in NCD and HFD groups. **j** Protein expression related to liver mitochondrial fission and fusion after surgery (left); quantitative results of mitochondrial fission and fusion related proteins (right). **k** TEM results of liver mitochondria in HFD-Sham group and HFD-SG group. **l-q** Mitochondrial area, mitochondrial circularity, ratio of mitochondrial area to cytoplasmic area, mitochondrial perimeter, mitochondrial aspect ratio, number of mitochondria per unit area in liver of HFD-Sham and HFD-SG groups. **r** Mitochondrial ETC complexes related protein expression at 16 weeks of feeding (left) and after surgery (right). **s** and **t** Quantitative results of mitochondrial ETC complexes. *N* = 6 per group per time point in all assays. The statistical data for all panels were presented as the mean ± SD. ∗ *p* < 0.05, ∗∗ *p* < 0.01, ∗∗∗ *p* < 0.001

Based on the aforementioned bioinformatic analyses, we identified MFF as a key candidate molecule linking MASLD to mitochondrial dysfunction. As a crucial mitochondrial outer membrane receptor for dynamin-related protein 1 (DRP1), MFF plays a central role in regulating the balance of mitochondrial fission and fusion dynamics. To validate the functional role of MFF in MASLD pathogenesis, we systematically assessed its expression dynamics and its impact on mitochondrial ultrastructure and ETC function in an animal model. Western blotting showed that at week 5, DRP1 and MFF showed a decreasing trend (Fig. 4a). However, after 16 weeks of HFD feeding, DRP1 and MFF were significantly upregulated, whereas mitochondrial fusion protein mitofusin2 (MFN2), mitochondrial fission protein 1 (FIS1), and uncoupling protein 2 (UCP2) were unchanged (Fig. 4b), indicating a biphasic temporal pattern. Transmission electron microscopy (TEM) revealed pronounced mitochondrial structural abnormalities in HFD-fed mice (Fig. 4c). Compared with NCD controls, mitochondrial area increased by approximately 80%, perimeter by 30%, circularity by 10%, and mitochondrial area/cytoplasmic area by 30%, whereas aspect ratio decreased significantly (Fig. 4d-i), consistent with mitochondrial swelling and fragmentation.

We further examined the regulatory role of bariatric surgery on the MFF-DRP1 axis and mitochondrial structure. SG reversed HFD-induced DRP1 and MFF upregulation without significantly affecting FIS1, MFN2, or UCP2 (Fig. 4j). TEM showed recovery toward an elongated mitochondrial morphology, with area and perimeter reduced by approximately 50%, lower circularity, and approximately 20% recovery of aspect ratio and mitochondrial density (Fig. 4k-q).

Given that mitochondrial respiratory function relies on OXPHOS mediated by ETC complexes, we finally assessed the protein expression of key ETC components. HFD feeding also reduced ETC complexes II, IV, and V by approximately 20%, 40%, and 50%, respectively (Fig. 4r, s), whereas SG increased complexes I, II, and IV (Fig. 4t). These findings linked HFD-induced MFF dysregulation to mitochondrial structural and respiratory abnormalities that were reversed by SG.

### Targeted downregulation of hepatic MFF largely mimicked the ability of SG to ameliorate MASLD phenotypes and restore mitochondrial homeostasis

**Fig. 5:**
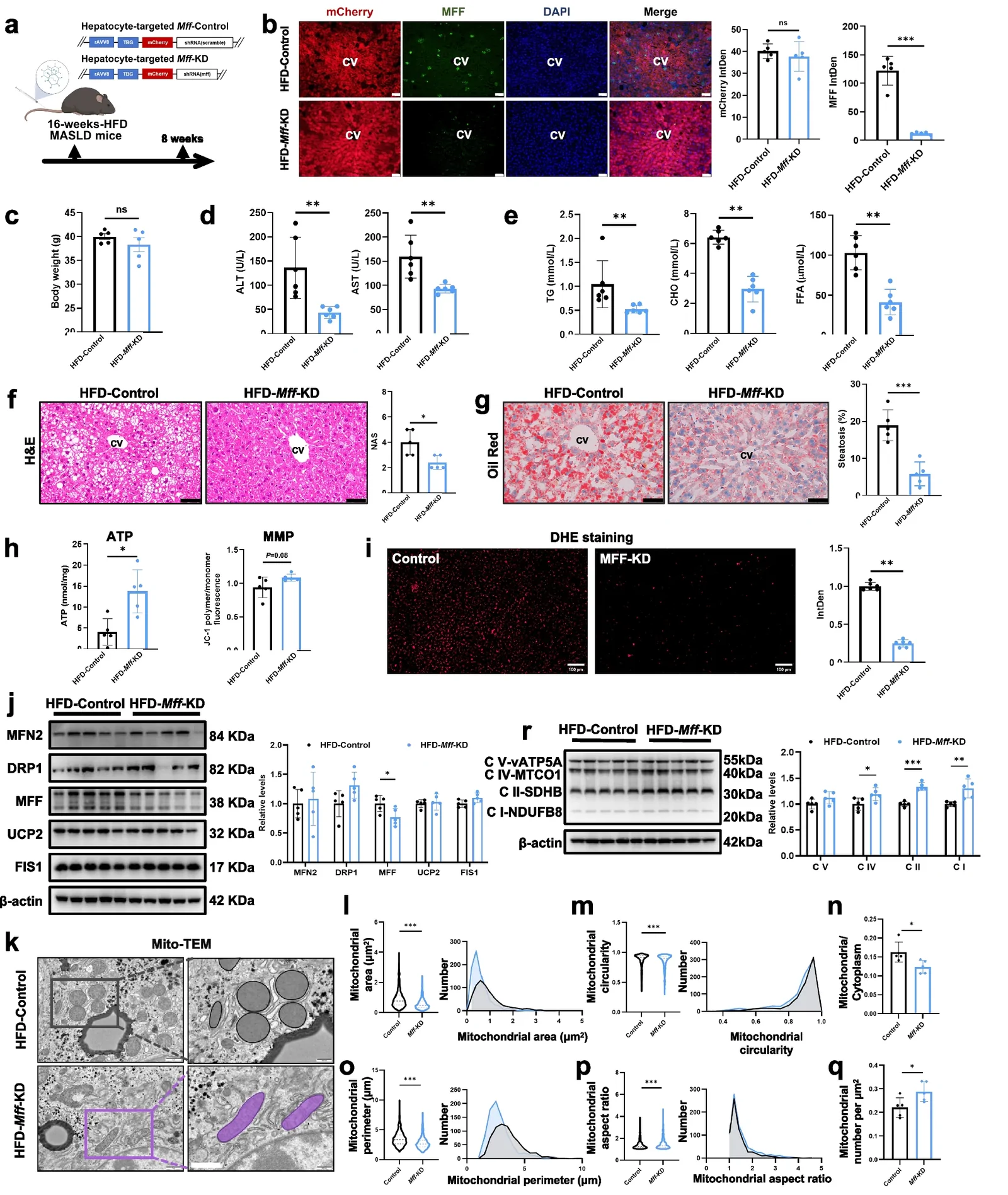
Down-regulating *Mff* in vivo simulated the surgical effect, improved the MASLD phenotype and restored the structure and function of mitochondria. **a** Schematic diagram of hepatocyte-specific *Mff*-KD AAV injection. **b** IF of MFF expression 8 weeks after AAV injection (scale bar: 50 μm) (left); mean fluorescence intensity of MFF (right). CV represented the central vein. **c** Change in body weight at 8 weeks after AAV injection. **d** ELISA results for serum ALT, AST at 8 weeks after AAV injection. **e** ELISA results for serum TG, CHO and FFA at 8 weeks after AAV injection. **f** Liver H&E staining (left) at 8 weeks after AAV injection (scale bar: 100 μm) and NAS scores (right). CV represented the central vein. **g** Liver oil red O staining (left) at 8 weeks after AAV injection (scale bar: 100 μm) and percentage of hepatic steatosis (right). CV represented the central vein. **h** Hepatic ATP and MMP levels at 8 weeks after AAV injection. **i** DHE staining of liver tissue sections at 8 weeks after surgery (scale bar: 100 μm). **j** Expression of hepatic mitochondrial fission and fusion related proteins at 8 weeks after AAV injection (left) and quantitative results (right). **k** TEM of hepatocyte mitochondria at 8 weeks after AAV injection. **l-q** Mitochondrial area, mitochondrial circularity, ratio of mitochondrial area to cytoplasmic area, mitochondrial perimeter, mitochondrial aspect ratio, number of mitochondria per unit area in liver of Control and *Mff*-KD groups. **r** Liver mitochondrial ETC complexes-related protein expression at 8 weeks after AAV injection and quantitative results. *N* = 5 per group per time point for all assays except serum biochemical analyses (**d**, **e**, **i**), where *N* = 6 per group. The statistical data for all panels were presented as the mean ± SD. ∗ *p* < 0.05, ∗∗ *p* < 0.01, ∗∗∗ *p* < 0.001

To determine whether MFF mediates the hepatic effects of SG, HFD-fed mice received hepatocyte-specific adeno-associated virus (AAV) serotype 8-mediated *Mff* knockdown (KD) after 16 weeks of HFD feeding (Fig. 5a). Eight weeks later, comparable mCherry reporter signals confirmed similar transduction efficiency, while hepatic MFF expression was reduced approximately six-fold (Fig. 5b). *Mff* mRNA and protein were selectively reduced in liver but not heart, gastrocnemius, stomach, or epididymal white adipose tissue (eWAT), confirming liver specificity (Fig. S6a, b). *Mff*-KD did not alter body weight but significantly reduced serum ALT, AST, TG, CHO, and FFA levels, with improvements comparable to those achieved by SG (Fig. 5c-e). Furthermore, *Mff*-KD enhanced glucose tolerance and insulin sensitivity in the mice (Fig. S6c, d). H&E and Oil Red O staining showed reduced steatosis, inflammatory infiltration, and ballooning degeneration; NAS fell to approximately 50% of control values, and hepatic LD area to approximately 30% (Fig. 5f, g). These results indicated that downregulating *Mff* effectively mimicked the metabolic protective effects of SG in vivo. *Mff*-KD also reduced the HFD-induced elevation of serum total bile acids (TBA) (Fig. S6e), suggesting that hepatic MFF downregulation modulated bile acid homeostasis.

*Mff*-KD increased ATP levels approximately fourfold and reduced ROS accumulation without significantly changing MMP (Fig. 5h, i). Expression of other mitochondrial dynamics-related proteins was unchanged (Fig. 5j). TEM showed restoration of elongated, rod-like mitochondria with more distinct cristae, accompanied by decreased mitochondrial area, circularity, perimeter, and mitochondrial area/cytoplasmic area and an increased aspect ratio; mitochondrial density was unchanged (Fig. 5k-q). Additionally, *Mff*-KD increased the protein expression of ETC complexes I, II, and IV by approximately 20% (Fig. 5r), indicating effective restoration of oxidative phosphorylation function.

To further confirm the role of MFF in mediating the therapeutic effects of SG, mice fed a HFD for 16 weeks were injected via the tail vein with *Mff* OE AAV and subsequently underwent SG (Fig. S7a, b). *Mff*-OE did not affect postoperative weight loss but partially attenuated SG-induced improvements in liver function, systemic lipid metabolism, hepatic steatosis, mitochondrial function, MFF-PLIN5 downregulation, and mitochondrial fission-related protein normalization (Fig. S7c-h). These results indicated that *Mff* OE partially abrogated the therapeutic effects of SG.

In summary, in vivo experiments confirmed that *Mff* downregulation significantly alleviated MASLD related metabolic disturbances and restored mitochondrial structure and respiratory function. The phenotypic improvements closely resembled those achieved by SG, suggesting that *Mff* was a key downstream target through which bariatric surgery regulated mitochondrial homeostasis and ameliorated hepatic lipid metabolism.

### In vitro studies demonstrated that *MFF* downregulation enhanced hepatocyte adaptation to high-fat stress and promoted mitochondrial fatty acid β-oxidation

**Fig. 6:**
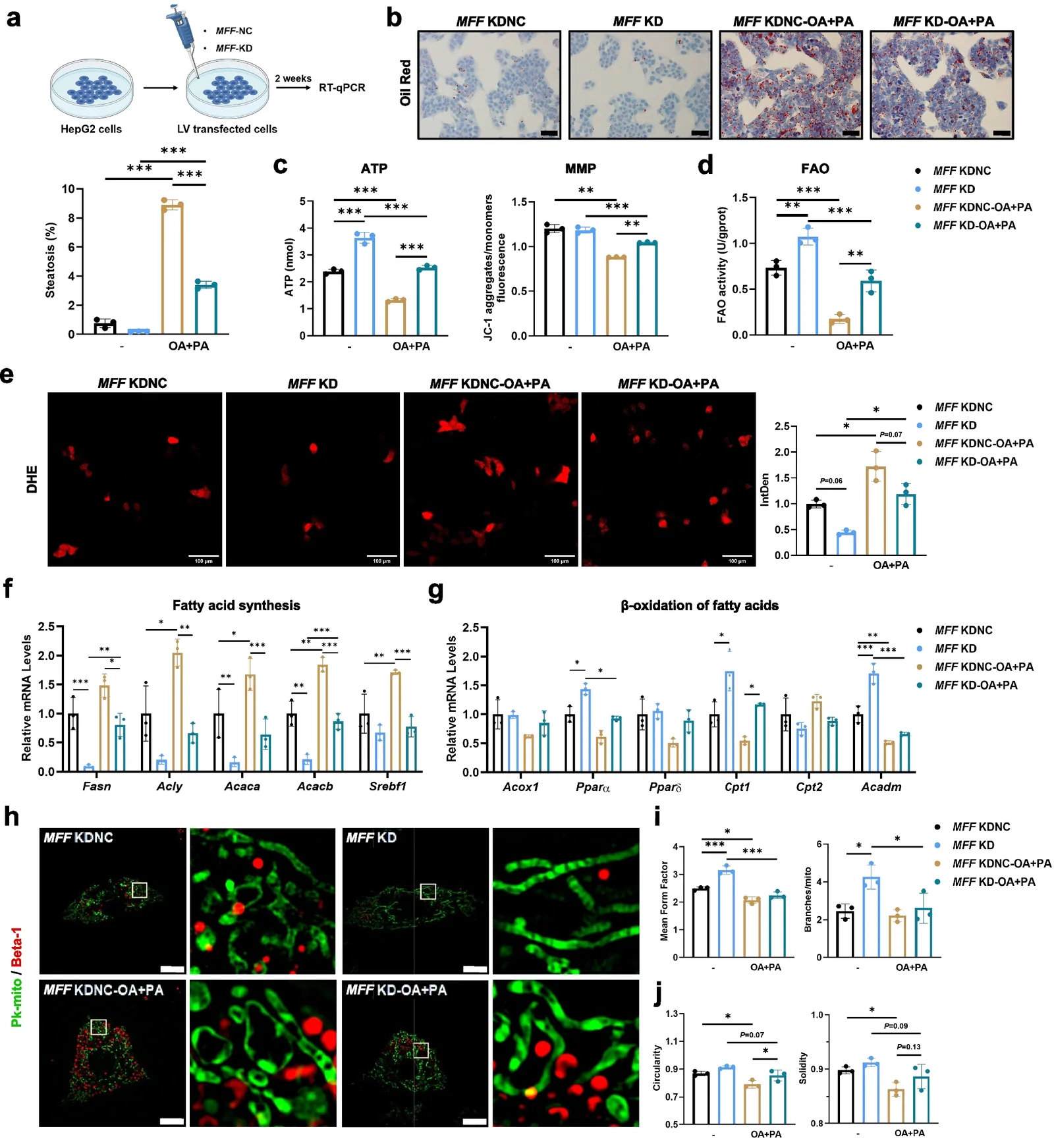
Down-regulating MFF enhanced the adaptability of mitochondria to high-fat stimulation and promoted fatty acid β-oxidation. **a** Schematic diagram of *MFF*-KD lentivirus transfection. **b** Oil Red O staining images (scale bars: 50 μm) and the calculated steatosis percentage. **c** ATP and MMP levels at 2 weeks after lentivirus transfection. **d** FAO activity levels at 2 weeks after lentivirus transfection. **e** DHE (ROS fluorescent probe) staining of HepG2 cells (scale bar: 100 μm). Quantitative results of mean fluorescence intensity. **f** and **g** Expression of synthesis and β-oxidation of fatty acid mRNA levels. **h** HIS-SIM images of mitochondria (Pk mito) and lipid droplets (Beta-1) (scale bars: 10 μm). **i** Quantitative results of HIS-SIM images about Mean Form Factor, Branches/mito. **j** Quantitative results of HIS-SIM images about Circularity and Solidity. “-” represented no OA and PA, “OA + PA” represented treatment with OA (660 μM) and PA (330 μM) for 48 h. *N* = 3 per group for all assays. The statistical data for all panels were presented as the mean ± SD. ∗ *p* < 0.05, ∗∗ *p* < 0.01, ∗∗∗ *p* < 0.001

To further validate the function of MFF and explore its mechanism of action, we established a stable *MFF*-KD cell model (Fig. 6a). RT-qPCR and Western blot confirmed effective suppression of MFF at both mRNA and protein levels, with expression reduced by approximately 50% (Fig. S8a, b). *MFF*-KD reduced LD accumulation and hepatocellular injury under OA + PA stimulation (Fig. 6b and Fig. S8c). Unlike the in vivo model, cells were subjected to *MFF* knockdown before OA + PA exposure to assess stress adaptation. Under high-fat conditions, ATP production and MMP remained at normal levels with no significant difference from controls (Fig. 6c), indicating that *MFF*-KD enhanced mitochondrial resilience to lipotoxic stress and helped maintain energy metabolic homeostasis.

*MFF*-KD increased FAO capacity by approximately 40% without excessive ROS accumulation and enhanced mitochondrial respiratory capacity by OCR analysis (Fig. 6d, e and Fig. S8d). It downregulated fatty acid synthesis genes while upregulating Pparα and its β-oxidation targets Cpt1 and Acadm (Fig. 6f, g). Similar transcriptional changes were observed in *Mff*-KD liver tissues (Fig. S8e, f). These findings suggested that *MFF* downregulation reprogrammed hepatocellular lipid metabolism by inhibiting lipid synthesis and promoting fatty acid oxidation. Super-resolution microscopy showed that *MFF*-KD preserved mitochondrial area and aspect ratio under high-fat stimulation and improved Mean Form Factor and branch number (Fig. 6h, i and Fig. S8g), indicating that *MFF*-KD helped preserve mitochondrial network integrity and mitigated high-fat-induced fragmentation and functional impairment. Concurrently, LDs exhibited increased circularity and solidity (Fig. 6j and Fig. S8h), suggesting reduced LD quantity and more structurally stable nascent LDs, consistent with enhanced metabolic clearance.

Conversely, stable *MFF*-OE increased lipid accumulation and impaired mitochondrial function (Fig. S9a-e), promoted fatty acid synthesis gene expression, suppressed β-oxidation-related genes, and reduced long-chain fatty acid (LCFA) oxidation measured by OCR (Fig. S9f, g). These results indicated that MFF downregulation exerted a protective effect against high-fat-induced mitochondrial dysfunction.

### Pathological remodeling of mitochondria-LD contact occurred in MASLD, SG and MFF downregulation reversed this aberrant interaction

**Fig. 7:**
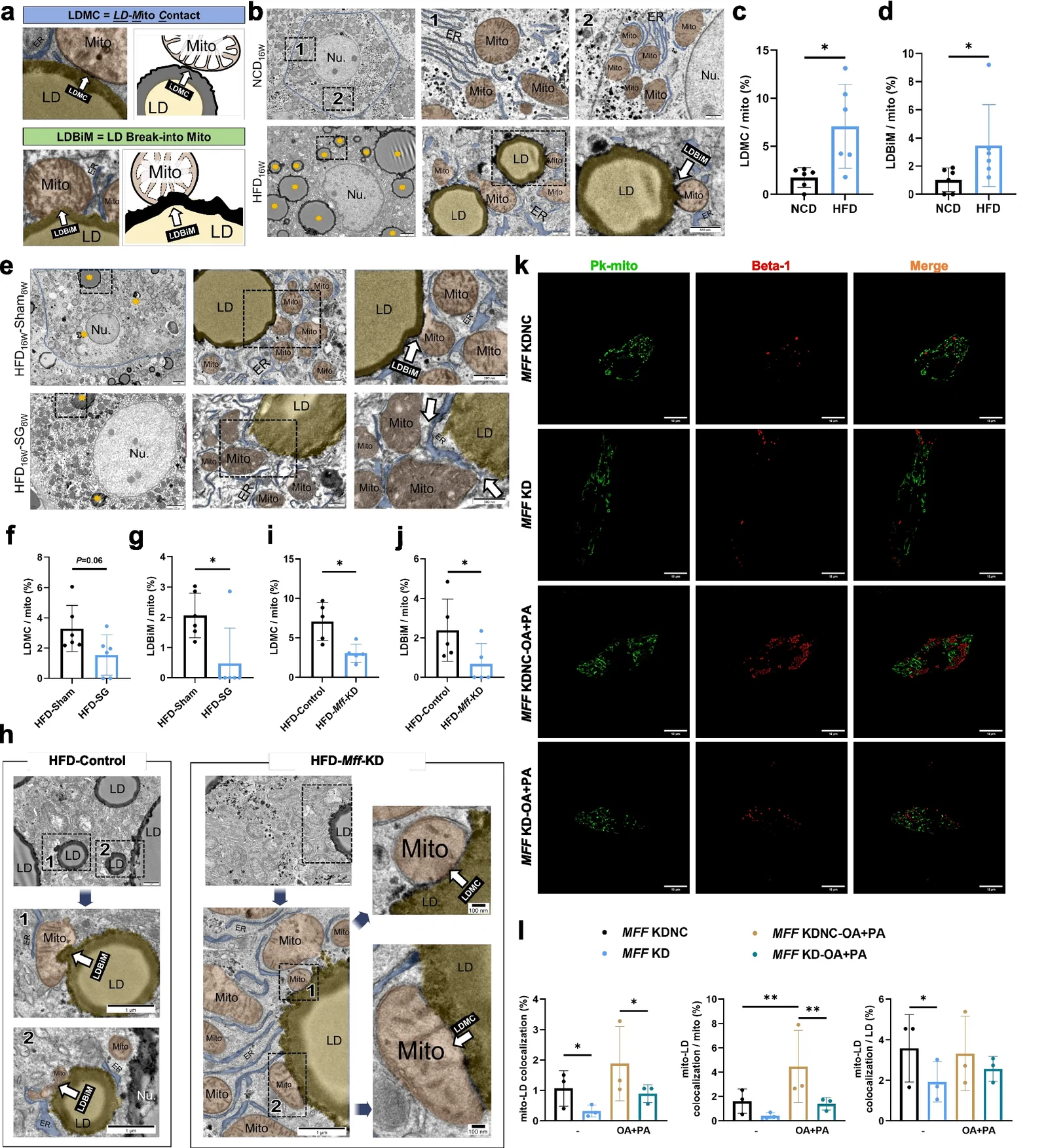
The mitochondrial-LD contact pattern in MASLD underwent pathological remodeling, which was reversed by SG and MFF-KD. **a** Schematic diagram of LDMC and LDBiM. **b** TEM of hepatocyte mitochondria after 16 weeks of feeding. **c** and **d** The percentage of LDMC and LDBiM in hepatocyte. **e** TEM of hepatocyte mitochondria at 8 weeks after surgery. **f** and **g** The percentage of LDMC and LDBiM in hepatocyte. **h** TEM of hepatocyte mitochondria at 8 weeks after AAV injection. **i** and **j** The percentage of LDMC and LDBiM in hepatocyte. **k** HIS-SIM images of mitochondria (Pk mito) and lipid droplets (Beta-1) (scale bars: 10 μm). **l** Quantitative results of HIS-SIM images about mito-LD colocalization. The statistical data for all panels were presented as the mean ± SD. ∗ *p* < 0.05, ∗∗ *p* < 0.01

Based on the aforementioned results that SG surgery and *Mff*-KD significantly improved mitochondrial structure and function, we further hypothesized that the contact interface between LDs and mitochondria, as a central hub of cellular lipid metabolism, may undergo functional and structural remodeling during MASLD progression. To explore its ultrastructural characteristics, we analyzed liver tissues using TEM and categorized mitochondria-LD interactions into two distinct patterns based on their spatial relationship: (1) LD-mitochondrion contact (LDMC): LDs and mitochondria were spatially adjacent or in direct contact, with a membrane distance less than 50 nm and intact mitochondrial outer membrane, representing a stable, non-invasive spatial association; (2) LD break-into mitochondria (LDBiM): LDs penetrated the mitochondrial outer membrane, intruding a distance of at least 100 nm, accompanied by disrupted cristae structure or outer membrane damage, indicating an abnormal and potentially destructive invasive interaction (Fig. 7a).

In NCD liver, mitochondria and LDs were generally small and spatially separated, and LDMC was the predominant interaction pattern. In HFD-induced MASLD, the proportion of LDBiM increased threefold, with LDs penetrating deeply into mitochondria and accompanied by membrane disruption and cristae loss (Fig. 7b-d). This structural alteration suggested that the pathological state of MASLD drove a remodeling of the mitochondria-LD interface from physiological proximity to pathological penetration. Both SG and hepatocyte-specific *Mff*-KD significantly reduced LDBiM events (Fig. 7e-j). Consistently, *MFF*-KD reduced mitochondria-LD colocalization and restored a more physiological contact pattern in vitro (Fig. 7k, l).

These findings indicated that restoring the physiological architecture of the mitochondria-LD interface was a crucial mechanism through which SG and MFF downregulation ameliorated MASLD phenotypes. These results uncovered a previously underappreciated ultrastructural pathological feature of MASLD and raised a key question: what molecular mechanism drives the formation of LDBiM?

### Elevated PLIN5 drove pathological contact and disrupted mitochondria-LD metabolic homeostasis

**Fig. 8:**
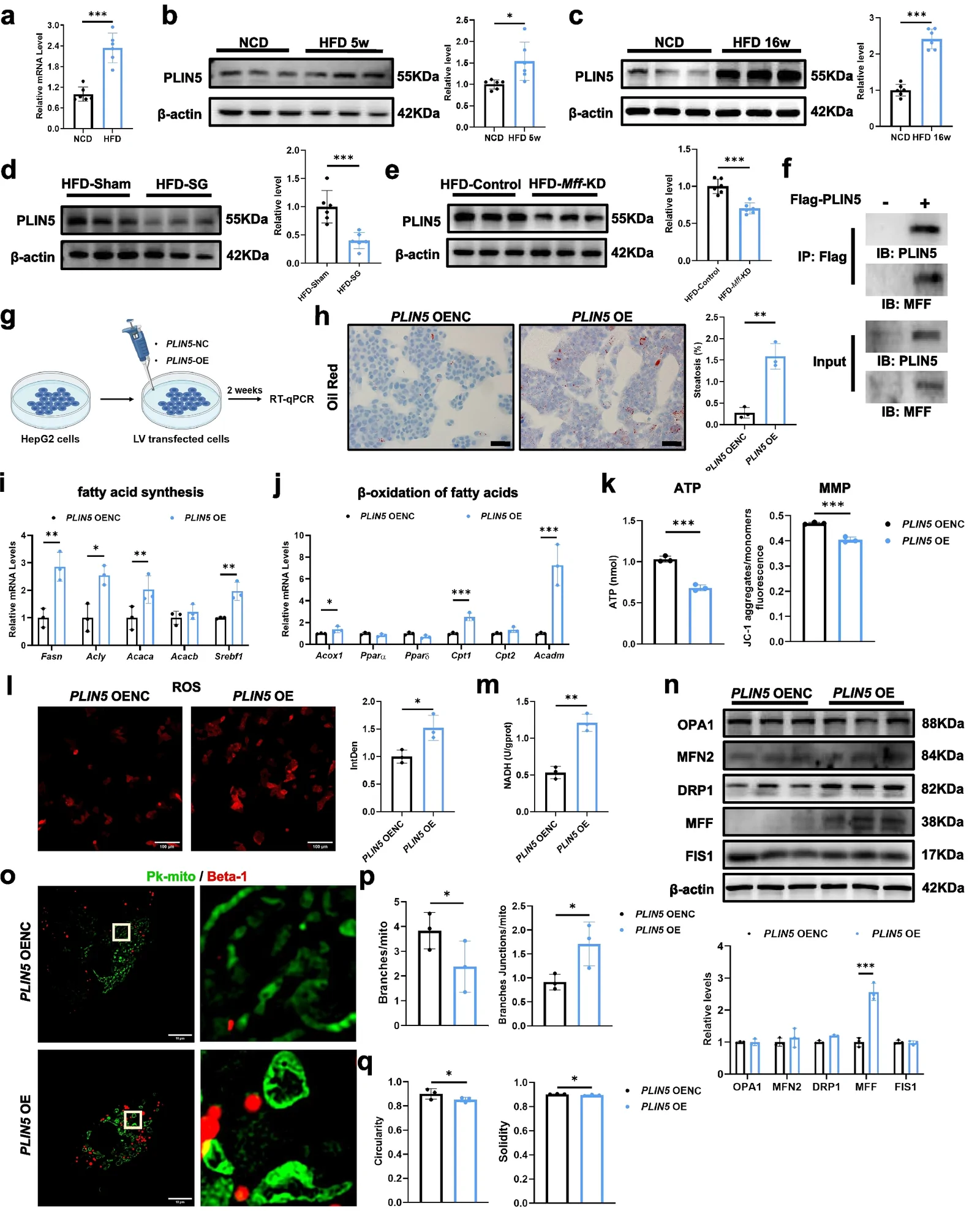
Abnormally elevated PLIN5 level drove pathological contact and led to metabolic imbalance of mitochondria and LDs. **a** Expression of *PLIN5* mRNA level after 16 weeks of HFD feeding. **b** Expression of PLIN5 protein level after 5 weeks of HFD feeding (left) and quantitative results (right). **c** Expression of PLIN5 protein level after 16 weeks of HFD feeding (left) and quantitative results (right). **d** Expression of PLIN5 protein level at 8 weeks after surgery (left) and quantitative results (right). **e** Expression of PLIN5 protein level at 8 weeks after AAV injection (left) and quantitative results (right). **f** The interaction between PLIN5 and MFF was examined in HepG2 cells transfected with PLIN5-3xFlag. **g** Schematic diagram of *PLIN5*-OE lentivirus transfection. **h** Oil Red O staining (scale bar: 50 μm) at 2 weeks after lentivirus transfection (left) and steatosis percentage (right). **i** and **j** Expression of synthesis and β-oxidation of fatty acid mRNA levels. **k** ATP and MMP levels at 2 weeks after lentivirus transfection. **l** DHE (ROS fluorescent probe) staining of HepG2 cells (scale bar: 100 μm). Quantitative results of mean fluorescence intensity. **m** NADH levels at 2 weeks after lentivirus transfection. **n** Expression of hepatic mitochondrial fission and fusion related proteins at 2 weeks after lentivirus transfection (up) and quantitative results (down). **o** HIS-SIM images of mitochondria (Pk mito) and lipid droplets (Beta-1) (scale bars: 10 μm). **p** and **q** Quantitative results of HIS-SIM images about Branches/mito, Branches Junctions/mito, circularity and solidity. *N* = 3 per group for all assays. The statistical data for all panels were presented as the mean ± SD. ∗ *p* < 0.05, ∗∗ *p* < 0.01, ∗∗∗ *p* < 0.001

To address the molecular mechanism driving the formation of LDBiM, we focused on PLIN5, a lipid droplet-associated protein known to play a central role in regulating LD stability and fatty acid mobilization [25]. PLIN5 is a strong candidate for mediating mitochondria-LD interactions [19, 26], yet its expression pattern in MASLD and its functional relationship with MFF remain unclear. During HFD feeding, PLIN5 mRNA and protein progressively increased, reaching approximately twofold higher levels by week 16, in contrast to the biphasic MFF expression pattern (Fig. 8a-c). Notably, both SG and *MFF*-KD intervention effectively reversed the abnormal upregulation of PLIN5, reducing its expression by approximately 60% and 40%, respectively (Fig. 8d, e). These findings suggested that PLIN5 was involved in MASLD pathogenesis and that its expression was regulated by MFF. To investigate a potential direct interaction between MFF and PLIN5, we performed co-immunoprecipitation (Co-IP) experiments. Results confirmed a specific binding interaction between MFF and PLIN5 in hepatocytes (Fig. 8f), providing direct molecular evidence for their collaborative role in lipid metabolism regulation.

Stable *PLIN5* overexpression (*PLIN5*-OE) in HepG2 cells increased LD size and number and induced hepatocellular injury (Fig. 8g, h and Fig. S10a-c). *PLIN5*-OE upregulated genes involved in both fatty acid synthesis and β-oxidation (Fig. 8i, j), but ATP production decreased by approximately 30%, MMP declined, and ROS increased (Fig. 8k-l). OCR analysis confirmed impairment of basal and maximal respiratory capacity (Fig. S10d). To functionally validate the changes in fatty acid flux, we performed BODIPY FL C16 fatty acid uptake staining and measured LCFA oxidation capacity. Increased fatty acid uptake at both 0 and 24 h, whereas maximal LCFA oxidation capacity did not increase proportionally (Fig. S11a, b and Fig. S12a), demonstrating an imbalance between fatty acid influx and oxidative capacity. This mismatch between fatty acid influx and oxidative capacity was accompanied by increased NADH levels (Fig. 8m) and elevated DRP1 and MFF expression (Fig. 8n).

Super-resolution imaging further showed mitochondrial swelling, reduced branching, and impaired network integrity after *PLIN5*-OE, although branch junction number increased. LD circularity and solidity decreased (Fig. 8o-q and Fig. S12b, c). *PLIN5*-OE also increased mitochondria-LD colocalization approximately threefold (Fig. S12d, e). Spatially, this explained the pathogenic mechanism: *PLIN5*-OE forced LDs into abnormally tight contact with mitochondria, delivering excessive fatty acids and ultimately causing mitochondrial metabolic overload and dysfunction.

This study delineated a pathological pathway in MASLD: dysregulated expression of MFF and PLIN5 led to excessive PLIN5-driven mitochondria-LD interactions, which overloaded fatty acid flux beyond mitochondrial oxidative capacity, triggering oxidative stress and functional collapse. SG counteracted this vicious cycle by downregulating MFF and normalizing PLIN5 expression, thereby restoring physiological balance at the mitochondria-LD interface. This intervention enhanced mitochondrial stability under MASLD conditions, improved mitochondrial function, reduced fatty acid synthesis, and promoted fatty acid β-oxidation, ultimately ameliorating lipid metabolic disturbances and alleviating MASLD.

## Discussion

This study elucidated the critical role of mitochondrial structural and functional integrity, as well as inter-organelle interactions, in maintaining lipid metabolic homeostasis. Our findings demonstrated that the upregulation of MFF and PLIN5 expression in mice subjected to long-term HFD feeding is a key event in MASLD progression. The MFF-PLIN5 axis mediated a shift in mitochondria-LD contact from physiological proximity LDMC to pathological penetration LDBiM, leading to mitochondrial fatty acid overload, oxidative stress, and functional collapse. Bariatric surgery, by downregulating MFF and PLIN5 expression, significantly reduced LD accumulation and its detrimental effects on mitochondrial integrity in MASLD mice, thereby optimizing mitochondrial structure and functional homeostasis and restoring hepatocyte lipid metabolism to alleviate MASLD symptoms. These results offered new insights into the pathogenesis of MASLD and suggest the MFF-PLIN5 axis as a potential therapeutic target.

The beneficial effects of SG on MASLD are generally attributed to both weight-dependent and weight-independent mechanisms [4, 27–29]. Weight loss reduces adipose-derived FFA influx, hepatic de novo lipogenesis, and insulin resistance [30, 31], whereas changes in gut hormones, bile acids, the gut microbiota, and inflammatory signaling may provide additional metabolic benefits [32–36]. The long-term resolution of MASH and regression of fibrosis after bariatric surgery further indicate that its effects extend beyond body-weight reduction [31, 37]. Consistent with this concept, our caloric-restriction experiments showed that SG produced greater improvements in hepatic steatosis, mitochondrial function, and MFF-PLIN5 expression than caloric restriction alone. Thus, SG may directly remodel intracellular lipid handling and mitochondrial homeostasis in hepatocytes.

MASLD develops through interactions among lipid overload, insulin resistance, oxidative stress, and organelle dysfunction. Hepatic TG accumulation is primarily driven by non-esterified fatty acid (NEFA) overflow from adipose tissue [38], coupled with increased de novo lipogenesis in the liver [39, 40]. When the liver’s capacity for lipid oxidation and export becomes overwhelmed [41, 42], toxic lipid intermediates, oxidative stress, and inflammatory responses intensify, ultimately driving the progression from simple steatosis to steatohepatitis and fibrosis [43, 44]. Although the classical “two-hit” model has been replaced by the “multiple-hit” concept [45], mitochondrial dysfunction remains an important determinant of disease progression [46–50]. Mitochondrial responses to lipid excess are also stage-dependent: early compensatory enhancement may gradually transition to decompensated dysfunction as lipotoxic stress accumulates [51–53]. The biphasic MFF pattern observed in our model, with early reduction followed by marked late-stage upregulation, may reflect this transition.

Targeted hepatic MFF downregulation largely recapitulated the protective effects of SG in vivo and in vitro, improving hepatic injury, lipid accumulation, mitochondrial morphology, ETC activity, and FAO. MFF is a mitochondrial outer-membrane receptor that recruits DRP1 to mitochondrial fission sites [54, 55]. Previous studies have shown that inhibiting DRP1 alleviated hepatic steatosis, oxidative stress, and tissue injury, and improved obesity-related metabolic deterioration [56, 57]. Moreover, under conditions of nutrient excess, mitochondrial fission has been implicated as a key mechanism driving ROS overproduction and cell death [58–61]. It should be noted, however, that existing literature on the role of MFF in fatty liver disease is not entirely consistent. While several studies support the notion that inhibiting mitochondrial fission alleviates hepatic steatosis and metabolic dysfunction [56, 57, 62, 63], other reports indicate that complete hepatocyte-specific knockout of MFF leads to aberrant mitochondrial morphology, endoplasmic reticulum stress, impaired TG secretion, and exacerbated MASH phenotypes under HFD conditions [64]. This detrimental effect may stem from defective mitophagy due to fission impairment, which has been shown to be partially rescued by simultaneous OPA1 knockdown to induce mitochondrial quiescence [65]. Moreover, restoring OPA1 can improve mitochondrial morphology without necessarily reducing hepatic lipid accumulation [66]. These observations suggest that physiological mitochondrial dynamics must be maintained within an appropriate range. Our data therefore support pathological MFF upregulation, rather than MFF itself, as the detrimental event in MASLD. Partial MFF downregulation limited excessive fission while preserving mitochondrial adaptive capacity under lipid stress.

Recent studies have emphasized that obesity and nutrient excess induce profound structural reorganization of the endoplasmic reticulum (ER) and mitochondria, which are physically and functionally interconnected through mitochondria-associated ER membranes (MAMs) [67]. Specifically, ER-mitochondria contacts are increased in hepatocytes of obese mice, promoting calcium transfer from the ER to mitochondria, leading to mitochondrial calcium overload, increased oxidative stress, and impaired insulin signaling. These findings resonate with our observations that mitochondria-LD contacts in MASLD also undergo a transition from LDMC to LDBiM. This dysregulation of inter-organelle communication may represent a common mechanism underlying organelle dysfunction in metabolic diseases. Furthermore, mitochondrial dynamics play a central role in maintaining organellar structural integrity. Our study found that MFF is aberrantly upregulated in MASLD, leading to mitochondrial fragmentation and dysfunction, consistent with the mitochondrial network fragmentation observed in obesity and diabetes [21]. Notably, ER stress itself can induce mitochondrial fission, suggesting a complex interaction network among the ER, mitochondria, and lipid droplets.

Similarly, mitochondria-LD contacts are altered under lipid-overloaded conditions [68–71]. Cui et al. described dynamic and anchored forms of mitochondria-LD contact [72], and studies of PDM have shown that LD-associated mitochondria possess metabolic properties distinct from cytosolic mitochondria and can be remodeled with MASLD severity and exercise [19, 73]. Their physiological significance, however, remains context-dependent. Some studies suggest that LD-mitochondria contacts promote fatty acid transfer and β-oxidation [72, 74–77], whereas others indicate that LD-associated mitochondria favor pyruvate oxidation and lipid esterification, facilitating LD expansion and protection from lipotoxicity [19, 26, 78–80]. To capture this structural heterogeneity, we classified mitochondria-LD interactions according to contact depth into LDMC and LDBiM. In MASLD, LDBiM increased markedly and was associated with mitochondrial membrane and cristae damage, whereas SG and *MFF*-KD restored a more physiological interaction pattern. These observations suggest that the biological consequence of mitochondria-LD contact depends not only on contact frequency but also on contact architecture and severity.

PLIN5 provides a plausible molecular link between altered lipid handling and mitochondria-LD contact. PLIN5 is highly expressed in oxidative tissues and regulates LD stability, lipolysis, fatty acid utilization, and physical coupling between LDs and mitochondria [81–88]. Previous studies, however, have reported apparently divergent effects of PLIN5. Hepatocyte-specific PLIN5 deletion can promote hepatic TG accumulation and insulin resistance [89], whereas PLIN5 overexpression has been associated with increased hepatic steatosis [87, 90]. Similar lipid accumulation has been reported in the heart without necessarily causing severe functional deterioration [69, 70, 91]. In our study, PLIN5 overexpression increased both fatty acid synthesis- and β-oxidation-related gene expression, consistent with reports that PLIN5 can simultaneously support TG storage and oxidative metabolism [82, 92, 93]. Nevertheless, fatty acid uptake increased disproportionately to oxidative capacity, accompanied by NADH accumulation, oxidative stress, mitochondrial dysfunction, and enhanced mitochondria-LD colocalization. Thus, PLIN5 may function as a context-dependent metabolic regulator: moderate coupling may facilitate lipid handling, whereas excessive PLIN5 expression may drive substrate delivery beyond mitochondrial oxidative capacity and promote pathological LDBiM.

From this perspective, SG and MFF downregulation appear to restore metabolic homeostasis through two coordinated effects. First, suppression of lipid synthesis reduces the lipid burden imposed on hepatocytes. Second, restoration of mitochondrial network integrity and normalization of mitochondria-LD interactions improve mitochondrial adaptation to sustained lipid exposure. This interpretation is consistent with evidence that bariatric surgery enhances hepatic mitochondrial respiration and biogenesis [94]. The MFF-PLIN5 axis may therefore represent a surgically modifiable molecular node that connects mitochondrial dynamics with intracellular lipid trafficking and could provide a potential target for non-surgical treatment of MASLD.

Several limitations should be acknowledged in this study. The temporal and causal relationship between lipid dysregulation and mitochondrial injury remains unresolved, and rescue experiments will be required to determine which event predominates during MASLD progression and SG-mediated reversal. In addition, SG is a systemic intervention that alters gut hormones, bile acid metabolism, the microbiota, and systemic inflammation; whether these signals directly regulate hepatic MFF-PLIN5 axis remains unknown. Finally, our study focused mainly on hepatocytes, whereas the liver also contains Kupffer cells, hepatic stellate cells, and sinusoidal endothelial cells. The role of the MFF-PLIN5 axis in these non-parenchymal populations warrants further investigation.

In conclusion, this study demonstrated that SG alleviated MASLD by suppressing the MFF-PLIN5 axis, thereby reducing pathological mitochondria-LD contact, lowering lipid synthesis burden, and restoring mitochondrial adaptability under sustained high-fat stress. Using a combination of animal models, hepatocyte-specific genetic manipulation, multi-omics analysis, and ultrastructural examination, we demonstrated that the MFF-PLIN5 axis mediates the shift from physiological LDMC to pathological LDBiM, leading to mitochondrial dysfunction and metabolic imbalance. These findings suggest that mitochondrial dynamics and organelle contact architecture are closely intertwined with hepatic lipid homeostasis, and that the MFF-PLIN5 axis may represent a surgically modifiable target for developing non-surgical therapies for MASLD.

## Materials and methods

### Animals and diet

8-week-old male C57BL/6 J mice (The Jackson Laboratory, Bar Harbor, Maine, USA) were fed different diets according to the groups: the control groups were fed NCD, and the HFD groups were fed an HFD with 60% kcal derived from fat (D12492, 60% kcal Fat Diet, biopike, China) and were maintained on the same diet after the surgery. Mice were housed at 24–26 °C with a circadian rhythm of 12 h for ad libitum food and water intake. All animal protocols adhered to the regulations of the Animal Welfare Ethics Committee of the Air Force Military Medical University, and were performed in accordance with the Guidelines for the Care and Use of Laboratory Animals (license number IACUC-20190107).

### Cell culture

HepG2 cells were cultured at 37 °C in a 100% relative humidity incubator containing 5% CO₂. The cells were maintained in high-glucose DMEM (C11995500BT, Gibco, USA) supplemented with L-glutamine, 10% fetal bovine serum (FBS) (164,210–50, Procell, China), and 1% penicillin–streptomycin (PB180120, Procell, China). The medium was refreshed every two days. The HepG2 cell line was authenticated by short tandem repeat (STR) analysis and tested negative for mycoplasma contamination. To induce lipid accumulation, cells were treated with a mixture of OA and PA (KC006, Kunchuang Biotechnology, China) for 48 h.

### Lentiviral transfection

Lentiviral vectors were packaged and synthesized by Hanbio (China), for lentiviral details, please see Table S1. HepG2 cells in good condition were seeded in 6-well plates for lentiviral transduction. At 48 h after infection, GFP expression efficiency was preliminarily observed under a fluorescence microscope. The optimal multiplicity of infection (MOI) for HepG2 cells was determined to be 5. The screening concentration of puromycin was 2 μg/mL. Experiments were performed after two weeks of stable transfection.

### AAV injection

A total of 200 μl of AAV8 containing 1 × 10^12^ vector genomes (V.G.) was injected into the tail vein of mice. The shRNA sequence targeting mouse *Mff* (GenBank accession NM_029409.3) was 5’-GATCGTGGGTTACAGGAAATAA-3’. A scrambled shRNA sequence with no known target was used as a negative control. Both the experimental and control viruses expressed mCherry as a reporter to monitor transduction efficiency and were driven by the thyroxine-binding globulin (TBG) promoter for hepatocyte-specific expression. WPRE (Woodchuck Hepatitis Virus Posttranscriptional Regulatory Element) was included to enhance transgene expression. The viral constructs were: rAAV-TBG-mCherry-5’miR30a-shRNA (Mff)−3’miR30a-WPRE (experimental) and rAAV-TBG-mCherry-5’miR30a-shRNA (scramble)−3’miR30a-WPRE (control).

### Bariatric surgery

After 16 weeks of HFD feeding, SG was performed in the HFD-SG group, and the surgical approach was based on a previous literature [95], while some of the surgical steps were changed according to the conditions provided by the laboratory. In the HFD-Sham group, only the abdomen was opened and sutured, and anesthesia was maintained for the same duration. 10% sugar water was administered from 6 h postoperatively, and a liquid diet was started after 24 h. The normal HFD was started after three days, and the sugar water was replaced with normal drinking water.

### OCR assay

The OCR, reflecting mitochondrial respiratory function, was measured using the Seahorse XF24 Extracellular Flux Analyzer (Agilent, USA) according to the manufacturer's instructions. HepG2 cells were seeded in XF24 cell culture microplates at a density of 2.5 × 10^5^ cells/mL. The sensor cartridge was hydrated overnight with Seahorse XF Calibrant Solution. On the day of the assay, cells were washed and maintained in Seahorse XF Base Medium supplemented with glucose, pyruvate, and glutamine as specified. Mitochondrial stressors (oligomycin, FCCP, and rotenone/antimycin A) were loaded into the injection ports of the sensor cartridge. OCR was measured under basal conditions and following sequential inhibitor injections.

### Fatty acid uptake assay

Palmitate (PAL) was conjugated to BSA following the standard protocol from Seahorse Biosciences. BODIPY FL C_16_ (D3821, Thermo Fisher Scientific) and PAL were conjugated in a 0.1% defatted bovine serum albumin solution for 30 min at 37 °C. Cells were incubated with 0.5 µM BODIPY-PAL overnight and immediately, after 24 h stained with Beta‑1 (HY‑152,073, MCE, USA) or Pk‑mito (PKMDR‑1, Genvivo, China) for 30 min. Live cells images were acquired using the confocal microscopy (Leica stellaris 5).

### Fatty acid oxidation assay

LCFA oxidation capacity was measured using the Seahorse XF LCFA Substrate Oxidation Stress Test (Agilent, 103,672–100) according to the manufacturer's instructions, with etomoxir (40 μM) used to confirm FAO-specific respiration.

### TEM observation

Tissue blocks of 1mm^3^ size were taken from fresh liver tissue on ice, fixed with special electron microscope solution, and stored at 4℃ away from light. The fixed liver tissue was dehydrated with ethanol, embedded, polymerized, sectioned, and stained to prepare copper mesh sections for electron microscopy analysis. The copper mesh sections were placed under a JEM-2000EX scanning electron microscope and photographed. We analyzed the images after scanning and labeled the mitochondria, endoplasmic reticulum, and LDs. Based on the contact between mitochondria and lipid droplets, we subdivided PDM into LDMC and LDBiM. ImageJ (1.53c, National Institutes of Health, USA) was used to analyze mitochondrial morphology, including mitochondrial area, mitochondrial circularity, ratio of mitochondrial area to cytoplasmic area, mitochondrial perimeter, mitochondrial aspect ratio, and number of mitochondria per unit area of the liver.

### Super resolution microscopy observation

Super‑resolution imaging of mitochondrial and LD structures was performed using a commercial HIS‑SIM system (CSR Biotech, China). Mitochondria were stained with Pk‑mito (PKMDR‑1, Genvivo, China), and LDs were labeled with Beta‑1 (HY‑152,073, MCE, USA). Images were acquired with a 100 × oil‑immersion objective. Cells were seeded in confocal dishes (D35C4‑20‑1.5‑N, Cellvis, USA) and maintained in a live‑cell imaging chamber for real‑time observation.

### Bioinformatics analysis

We downloaded two human liver transcriptome sequencing datasets (GSE135251 and GSE106737) and one mouse liver single-cell sequencing dataset (GSE129516) from the GEO database. GSE135251 contains tissue sequencing data from 155 MASH and 10 normal livers. GSE106737 contains liver tissue sequencing data from 21 MASH patients before and after bariatric surgery. Differential gene analysis was performed using the DESeq2 package in GSE135251 to screen for MASH-associated DEGs. The screening criteria for DEGs were | log2 fold change |> 0.585 and *P* < 0.05. We used the limma package to analyze DEGs in GSE106737 to screen for differentially expressed genes related to bariatric surgery. The filtering criteria for DEGs were | log2 fold-change |> 0.263 and *P* < 0.05. Based on theses DEGs, GO/KEGG enrichment analysis was performed using the cluster Profiler package. The screening criterion of *P* < 0.05 was statistically significant.

The single-cell sequencing data were processed as follows: first, the data were read and Seurat objects were created; then, the data were filtered using the following criteria: nFeature_RNA > 500 & nFeature_RNA < 7500, nCount_RNA < 10,000, percent_mito < 25, percent_ribo > 3, percent_hb < 1. Subsequently, we integrated single-cell samples from the two datasets using Harmony. After integration, the Seurat objects were subjected to dimensionality reduction cluster clustering, a resolution of 0.5, and cell annotation was performed using the Single R package. Find Markers, a built-in function of Seurat V5, were used to identify DEGs of Hepatocytes in MASH and healthy groups.

### GSEA analysis

Patients were divided into two subgroups, high and low, based on the median value of HUB genes. The potential biological significance of HUB genes was studied by GSEA. Two predefined gene sets, namely "h.all.v2023.1.Hs.Symbols" and " c2.cp.kegg.v2023.1.Hs.Symbols", were downloaded from the Molecular Signatures Database (https://www.gsea-msigdb.org/gsea/msigdb/index.jsp). GSEA was performed using R packets “clusterProfiler.” The inclusion criteria were normalized enrichment > 1, adjusted *p* < 0.05, and false discovery rate < 0.25 [96].

### Statistical analysis

All statistical analyses were performed using GraphPad Prism 10 (GraphPad Software, La Jolla, California, USA). For comparisons between two groups, Student’s t-test (for parametric data) or the Mann–Whitney U test (for non-parametric data) was used to determine statistical significance. For multiple comparisons, two-way ANOVA followed by Tukey's post hoc test was applied. All Western blot and functional cellular assays were repeated at least three times to ensure reproducibility. Significance was represented by *p < 0.05, **p < 0.01, and ***p < 0.001. Data were presented as mean ± SD.

Additional experimental methods were described in the Supplementary Information.

## Supplementary Information

Supplementary Material 1.

## Data Availability

All data necessary for confirming the conclusions are included in this article. The datasets generated and/or analyzed during the current study are available from the author upon reasonable request.
