# Supplementary material for: Bariatric surgery may alleviate MASLD by restoring mitochondria-lipid droplet structural and functional integrity through suppression of the Mitochondrial Fission Factor-Perilipin 5 axis
Source: Mol Biomed. 2026 Oct 6;7:198. doi: 10.1186/s43556-026-00607-0 (PMC13638945; doi:10.1186/s43556-026-00607-0)
Supplement: Supplementary file 1 [file 43556_2026_607_MOESM1_ESM.docx]

**Supplementary Material**

**Title:**

Bariatric surgery may alleviate MASLD by restoring mitochondria-lipid droplet structural and functional integrity through suppression of the Mitochondrial Fission Factor-Perilipin 5 axis

**Authors:**

Yuze Sun^2†^, Yuxuan Liu^2†^, Xiyu Chen^1, 4†^, Shizhou Deng^1^, Ning Zhang^1^, Feifei Wu^3^, Shujiao Li^3^, Dong Fan^1^, Yayun Wang^3*^, Yanling Yang^1, 2*^

**Authors’ Affiliations:**

^1^Department of Hepatobiliary Surgery, Xi-Jing Hospital, Air Force Medical University, Xi’an, China.

^2^Department of General Surgery, Hepatobiliary Surgery Center, Tangdu Hospital, The Fourth Military Medical University, Xi’an 710038, China.

^3^National Demonstration Center for Experimental Preclinical Medicine Education, Air Force Medical University, Xi’an, China.

^4^Western Theater Command Air Force Hospital, Chengdu, China.

† These authors contributed to this work equally

*** Corresponding authors:**

Yanling Yang, MD, PhD

Email: yangyanl@fmmu.edu.cn; ORCID: 0000-0001-8246-1756

Office Tel: 0086-29-84712341

Mobile: 0086-13709246656

Yayun Wang, MD, PhD

Email: wangyy@fmmu.edu.cn; ORCID: 0000-0002-0397-0390

Office Tel: 0086-29-84774104

Mobile: 0086-13679168991

**Table S1:** **Lentivirus used in the study**

| Lentivirus | Groups | Vendor | Item No. | Batch No. | Lentivirus vector | Titer (TU/mL) |
| --- | --- | --- | --- | --- | --- | --- |
| *MFF* | sh-*MFF*-NC | Hanbio | HH20230822ZY-LV01 | LV83090134 | pHBLV-U6-MCS-CMV-ZsGreen-PGK-PURO | 1.5*10^8 |
| *MFF* | sh-*MFF* | Hanbio | HH20230822ZY-LV01 | LV83090132 | pHBLV-U6-MCS-CMV-ZsGreen-PGK-PURO | 1.5*10^8 |
| *MFF* | OE-*MFF*-NC | Hanbio | HH20230822ZY-LP01 | LV82082903 | pHBLV-CMV-MCS-3FLAG-EF1-ZsGreen-T2A-PURO | 2*10^8 |
| *MFF* | OE-*MFF* | Hanbio | HH20230822ZY-LP01 | LV82082904 | pHBLV-CMV-MCS-3FLAG-EF1-ZsGreen-T2A-PURO | 2*10^8 |
| *PLIN5* | OE-*PLIN5*-NC | Hanbio | HH20241227ZY-LVP01 | LV99010354 | HBLV-ZsGreen-PURO | 4.5*10^8 |
| *PLIN5* | OE-*PLIN5* | Hanbio | HH20241227ZY-LVP01 | LV99010355 | HBLV-h-Plin5-3xflag-ZsGreen-PURO | 4*10^8 |

**Table S2: RT-qPCR primer sequence**

| Name | Species | Primer sequence-F | Primer sequence-R |
| --- | --- | --- | --- |
| *FASN* | Human | TGTTTGGTGTTTGTCTGTGTTTGT | TGGAGATCACATGCGGTTTAATT |
| *ACLY* | Human | CCTCACGAGAAAGCTGATCAAGA | CCACCTGTGTTGCCAATCTTAAA |
| *ACACA* | Human | ACCTGCGAGTAGAGACACAATTC | CGGCCACACAACTGATCAATAAG |
| *ACACB* | Human | GGAGCTCGATGACCCTTCTAAAG | GCACCCACTCCTTCAGCTTTAT |
| *SREBF1* | Human | CATCTCTTAGAGCGAGCACTGAA | TGGAACTGATGGAGAAGCTGTAG |
| *ACOX1* | Human | CGCCGAGAGATCGAGAACAT | GACAGCCACCTCATAACGCT |
| *PPARα* | Human | CCTGTCTGCTCTGTGGACTC | CCGAGCTCCAAGCTACTCTT |
| *PPARδ* | Human | GGGAGAGCTACTGAGTTGAACTG | ACAGTTCCAACCCTTAGCAAGTA |
| *CPT1* | Human | GATCGTCACCTCTTCTGCCTTTA | CTGGACACGTACTCTGGGTTATT |
| *CPT2* | Human | TCGGGACCCTGGTTTGATATGTA | GCACAAAGCGTATGAGTCTCTTG |
| *ACADM* | Human | GAGCTGGTTTCAAAGTTGCAATG | TGCCATTTCAGCCAGCATAAAT |
| *MFF* | Human | AGATCTTGGATGTGCTGGATGAA | GCACGTTCTTTGTTCTCCTCTTC |
| *PLIN5* | Human | CTTCCCTTTCTCCAGCAACCTTC | TTCCAGTACAACATCCACAGCAT |
| *GAPDH* | Human | GATTCCACCCATGGCAAATTC | CTGGAAGATGGTGATGGGATT |
| *Fasn* | Mouse | TGCACCTCACAGGCATCAAT | GTCCCACTTGATGTGAGGGG |
| *Acly* | Mouse | CCCAAGATTCAGTCCCAAGTC | TTGTGATCCCCAGTGAAAGG |
| *Acaca* | Mouse | CGCCAGCCTGAGTTCTTT | GGCCAACGGAGATGGTTCAT |
| *Acacb* | Mouse | TGGAAACGCAGTTTGGAAGC | CAAGACCATTCAGGCAACTGTG |
| *Srebf1* | Mouse | CTTTTCCTTAACGTGGGCCT | GAGCTGGAGCATGTCTTCGAT |
| *Acox1* | Mouse | TTTGTGGAACCTGTTGGCCT | TCGAAGATGAGTTCCGTGGC |
| *Pparα* | Mouse | GTGCAGCCTCAGCCAAGT | GAGGACAGATGGGGCTCTCT |
| *Pparδ* | Mouse | AGTCATGGAACAGCCACAGG | GAATTCTGGGAGAGGTCTGCAC |
| *Cpt1* | Mouse | CATTCCGCCGCCGCC | ACCAGTGATGATGCCATTCTTG |
| *Cpt2* | Mouse | CCATGCACTACCAGGACAGC | ACACAACACTTCTGTCTTCCTGA |
| *Acadm* | Mouse | TGACAAAAGCGGGGAGTACC | GCACCCCTGTACACCCATAC |
| *Mff* | Mouse | ATGCCAGTGTGATAATGCAAGT | CTCGGCTCTCTTCGCTTTG |

**Table S3: Antibody information**

| Name | Species | Supplier | Catalog number | Dilution ratio |
| --- | --- | --- | --- | --- |
| UCP2 | R | Cell Signaling Technology | Cat#89326S | 1:1000 |
| DRP1 | R | Cell Signaling Technology | Cat#8570S | 1:1000 |
| FIS1 | R | Cell Signaling Technology | Cat#32525S | 1:1000 |
| MFF | R | Cell Signaling Technology | Cat#84580S | 1:1000 |
| MFN2 | R | Cell Signaling Technology | Cat#9482S | 1:1000 |
| PLIN5 | R | Proteintech | 26951-1-AP | 1:1000 |
| OPA1 | R | Abcam | ab184247 | 1:1000 |
| β-actin | M | Engibody | AT0001 | 1:1000 |
| OXPHOS Cocktail | M | Abcam | ab110413 | 1:250 |
| HRP | R | Abbkine | A21020 | 1:5000 |
| HRP | M | Abbkine | A21010 | 1:5000 |

**Fig. S1. HFD feeding induced obesity, liver injury, and glucose metabolic disorders in mice.**

(**a**) Body fat content and lean content after 16 weeks of feeding. (**b**) ELISA results for serum ALT, AST at four time points. (**c**) IPGTT and the calculated AUC at four time points. (**d**) IPITT and the calculated AUC at four time points.

N = 6 per group per time point in all assays. The statistical data for all panels were presented as the mean ± SD. ∗ *p* < 0.05, ∗∗ *p* < 0.01, ∗∗∗ *p* < 0.001.

**Fig. S2. OA+PA stimulation induced lipid accumulation and mitochondrial dysfunction in HepG2 cells.**

(**a**) Oil Red O staining (scale bar: 50 μm) at 48 hours after OA+PA (left) and steatosis percentage (right). (**b**) and **(c)** ATP and MMP levels at 48 hours after OA+PA. (**d**) DHE (ROS fluorescent probe) staining of MASLD cell model (scale bar: 100 μm). Quantitative results of mean fluorescence intensity. (**e**) FAO activity levels at 48 hours after OA+PA. **(f)** Seahorse XF Cell Mitochondrial Stress Test, statistical analysis of various indices representing the mitochondria OXPHOS ability of MASLD cell model. (**g**) and (**h**) Expression of synthesis and β-oxidation of fatty acid mRNA levels.

N = 3 per group for all assays. The statistical data for all panels were presented as the mean ± SD. ∗ *p* < 0.05, ∗∗ *p* < 0.01, ∗∗∗ *p* < 0.001.

**Fig. S3. HFD feeding altered hepatic fatty acid metabolism gene expression in vivo.**

(**a**) Expression of fatty acid synthesis mRNA levels. (**b**) Expression of fatty acid β-oxidation mRNA levels.

N = 6 per group for all assays. The statistical data for all panels were presented as the mean ± SD. ∗ *p* < 0.05, ∗∗ *p* < 0.01, ∗∗∗ *p* < 0.001.

**Fig. S4. SG reversed HFD-induced alterations in hepatic fatty acid metabolism gene expression.**

(**a**) Expression of fatty acid synthesis mRNA levels. (**b**) Expression of fatty acid β-oxidation mRNA levels.

N = 6 per group for all assays. The statistical data for all panels were presented as the mean ± SD. ∗ *p* < 0.05, ∗∗ *p* < 0.01, ∗∗∗ *p* < 0.001.

**Fig. S5. Caloric restriction partially improved MASLD phenotypes, but SG exerted superior effects independent of weight loss.**

(**a**) Schematic diagram of operation procedure. (**b**) Weight loss at 0-4 weeks after surgery and feeding. (**c**) ALT and AST at 4 weeks after surgery and feeding. (**d**) ELISA results for serum TG, CHO and FFA at 4 weeks after surgery and feeding. (**e**) Liver H&E staining (scale bar: 100 μm) at 4 weeks after surgery and feeding (left), NAS scores (right). (**f**) Liver Oil Red O staining (scale bar: 100 μm) at 4 weeks after surgery and feeding (left), steatosis percentage (right). (**g**) Liver ATP, MMP and ROS levels at 4 weeks after surgery and feeding. (**h**) Protein expression related to liver mitochondria and LDs at 4 weeks after surgery and feeding (left); quantitative results of these proteins (right).

N = 6 per group for all assays. The statistical data for all panels were presented as the mean ± SD. ∗ *p* < 0.05, ∗∗ *p* < 0.01, ∗∗∗ *p* < 0.001.

**Fig. S6. Liver-specific *Mff* knockdown was confirmed at the mRNA and protein levels, and improved glucose metabolism and bile acid homeostasis in vivo.**

(**a**) Expression of *Mff* mRNA level at 8 weeks after AAV injection. (**b**) Expression of MFF protein level at 8 weeks after AAV injection (left) and quantitative results (right). (**c**) IPGTT and their calculated AUC at 8 weeks after AAV injection (N=5). (**d**) IPITT and their calculated AUC at 8 weeks after AAV injection (N=5). (**e**) ELISA results for serum TBA at 8 weeks after AAV injection.

N = 6 per group for all assays. The statistical data for all panels were presented as the mean ± SD. ∗ *p* < 0.05, ∗∗ *p* < 0.01, ∗∗∗ *p* < 0.001.

**Fig. S7. *Mff* overexpression partially abrogated the therapeutic effects of SG in vivo.**

(**a**) Schematic diagram of the experimental timeline and surgical procedure. (**b**) Expression of *Mff* mRNA level at 4 weeks after AAV injection and surgery. (**c**) Weight loss at 0-4 weeks after AAV injection and surgery. (**d**) ALT and AST at 4 weeks after AAV injection and surgery. (**e**) ELISA results for serum TG, CHO and FFA at 4 weeks after AAV injection and surgery. (**f**) Liver Oil Red O staining (scale bar: 100 μm) at 4 weeks after AAV injection and surgery (left), steatosis percentage (right). (**g**) Liver ATP, MMP and ROS levels at 4 weeks after AAV injection and surgery. (**h**) Protein expression related to liver mitochondria and LDs at 4 weeks after AAV injection and surgery (left); quantitative results of these proteins (right).

N = 6 per group for all assays. The statistical data for all panels were presented as the mean ± SD. ∗ *p* < 0.05, ∗∗ *p* < 0.01, ∗∗∗ *p* < 0.001.

**Fig. S8. *MFF* knockdown enhanced mitochondrial respiratory capacity and altered fatty acid metabolism gene expression in vitro and in vivo.**

(**a**) Expression of *MFF* mRNA level at 2 weeks after lentivirus transfection. (**b**) Expression of MFF protein level at 2 weeks after lentivirus transfection (left) and quantitative results (right). (**c**) ALT and AST at 2 weeks after lentivirus transfection. **(d)** Seahorse XF Cell Mitochondrial Stress Test, statistical analysis of various indices representing the mitochondrial OXPHOS ability at 2 weeks after lentivirus transfection. (**e**) and (**f**) Expression of fatty acid synthesis and β-oxidation mRNA levels at 8 weeks after AAV injection. (**g**) and (**h**) Quantitative results of HIS-SIM images about Mean Area, Mean Aspect ratio, Branches Junctions/mito, Average Size and Mean Perimeter.

N = 3 per group for all assays. The statistical data for all panels were presented as the mean ± SD. ∗ *p* < 0.05, ∗∗ *p* < 0.01, ∗∗∗ *p* < 0.001.

**Fig. S9. *MFF* overexpression exacerbated lipid accumulation and impaired mitochondrial function in HepG2 cells.**

(**a**) Schematic diagram of *MFF*-OE lentivirus transfection. (**b**) Expression of *MFF* mRNA level at 2 weeks after lentivirus transfection. (**c**) Expression of MFF protein level at 2 weeks after lentivirus transfection (left) and quantitative results (right). **(d)** Oil Red O staining images (scale bars: 50 μm) and the calculated steatosis percentage. **(e)** ATP, MMP and ROS levels at 2 weeks after lentivirus transfection. (**f**) Expression of synthesis and β-oxidation of fatty acid mRNA levels at 2 weeks after lentivirus transfection. (**g**) Seahorse XF LCFA Substrate Oxidation Stress Test, statistical analysis of △OCR at 2 weeks after lentivirus transfection.

N = 3 per group for all assays. The statistical data for all panels were presented as the mean ± SD. ∗ *p* < 0.05, ∗∗ *p* < 0.01, ∗∗∗ *p* < 0.001.

**Fig. S10. *PLIN5* overexpression induced hepatocellular injury and impaired mitochondrial respiratory capacity in HepG2 cells.**

(**a**) Expression of *PLIN5* mRNA level at 2 weeks after lentivirus transfection. (**b**) Expression of PLIN5 protein level at 2 weeks after lentivirus transfection (left) and quantitative results (right). (**c**) ALT and AST at 2 weeks after lentivirus transfection. **(d)** Seahorse XF Cell Mitochondrial Stress Test, statistical analysis of various indices representing the mitochondrial OXPHOS ability at 2 weeks after lentivirus transfection.

N = 3 per group for all assays. The statistical data for all panels were presented as the mean ± SD. ∗ *p* < 0.05, ∗∗ *p* < 0.01.

**Fig. S11. *PLIN5* overexpression enhanced fatty acid uptake and mitochondria-LD colocalization.**

(**a**) HIS-SIM images of fatty acid (BODIPY-PAL) and lipid droplets (Beta-1), and quantitative results of fatty acid-LD colocalization. (**b**) HIS-SIM images of fatty acid (BODIPY-PAL) and mitochondria (Pk-mito), and quantitative results of fatty acid-mitochondria colocalization. Scale bars: 25 μm.

N = 3 per group for all assays. The statistical data for all panels were presented as the mean ± SD. ∗ *p* < 0.05, ∗∗ *p* < 0.01, ∗∗∗ *p* < 0.001.

**Fig. S12. *PLIN5* overexpression reduced fatty acid oxidation and disrupted mitochondrial-LD structural integrity.**

(**a**) Seahorse XF LCFA Substrate Oxidation Stress Test, statistical analysis of △OCR at 2 weeks after lentivirus transfection. (**b**) and (**c**) Quantitative results of HIS-SIM images about Mean Area, Mean Aspect Ratio, Mean Form Factor, Average Size and Mean Perimeter. **(d)** HIS-SIM images of mitochondria (Pk mito) and lipid droplets (Beta-1). **(e)** Quantitative results of HIS-SIM images about mito-LD colocalization.

N = 3 per group for all assays. The statistical data for all panels were presented as the mean ± SD. ∗ *p* < 0.05.

**Materials and methods:**

1. **IPGTT and IPITT**

IPGTT and IPITT were performed to explore glucose metabolism. IPGTT: Mice were injected intraperitoneally with 20% glucose (1 g/kg) after 6 h of fasting, and tail vein glucose values were measured immediately before and 15, 30, 60, 90, and 120 min after injection using a glucometer (Roche, Germany). IPITT: Mice were injected intraperitoneally with insulin (0.5/ U/kg) after 6 h of fasting, and tail vein blood glucose values were measured immediately before and 15, 30, 60, 90, and 120 min after injection.

2. **Component analysis**

Body fat and lean content in mice were analyzed after a specific diet and surgery using an animal component analyzer (BrukerLF90, Bruker, Germany).

3. **Serum analysis**

Mice were anesthetized by intraperitoneal injection of sodium pentobarbital (50 mg/kg). When they entered deep anesthesia, the chest cavity was opened, the heart was exposed, and blood was collected from the heart using a syringe. The Blood was allowed to rest overnight at 4°C, and serum was collected by centrifugation (3000 rpm, 4°C, 20 min). The serum was rapidly frozen in liquid nitrogen and stored at -80°C for subsequent biochemical tests. Serum glucose, ALT, AST, FFA, CHO and TG levels were measured using a biochemical index test kit (Redu, Changchun Huili, China), TBA were measured using colorimetric analysis kits (E-BC-K181-M, Elabscience, China).

4. **Histological analyses**

Liver specimens were fixed in 4% formalin buffer, paraffin-embedded, and serial 4-μm thick sections were used for H&E staining to evaluate hepatocyte morphology. The fixed liver specimens were subjected to frozen section Oil Red O staining to evaluate hepatic steatosis. Liver steatosis, activity, and lobular inflammation were scored semi-quantitatively according to the NAS.

5. **Oil Red O Staining of Cells**

Oil Red O powder was dissolved in isopropanol at a concentration of 0.2 g/mL and incubated at 65°C for 24 hours to prepare a saturated stock solution. The working solution was freshly prepared by diluting the stock solution with distilled water at a ratio of 3:2. After treatment, HepG2 cells were fixed with 4% paraformaldehyde and permeabilized, followed by a brief rinse with 60% isopropanol. Cells were then stained with Oil Red O working solution at room temperature for 10-20 minutes. After rapid washing with 60% isopropanol, nuclei were counterstained with hematoxylin.

6. **ROS assay**

Hepatic and cellular ROS levels were assessed using the Tissue ROS Assay Kit (BB-470512, Bestbio, China) and the DHE-ROS Detection Kit (BB-47051, BestBio, China), respectively, according to the manufacturer’s protocols.

For liver tissue: Fresh tissue samples were rinsed with Phosphate Buffered Saline (PBS). Approximately 50 mg of liver tissue was homogenized in 500 μL of homogenization buffer using a tissue homogenizer. The homogenate was centrifuged at 100 × g for 5 min at 4°C, and the supernatant was collected. A 190 μL aliquot of the supernatant was mixed with 10 μL of the O13 probe in a 96-well plate and incubated in the dark at 37°C for 20-30 min. Fluorescence intensity was measured at excitation/emission wavelengths of 488/606 nm using a SpectraMax M5 microplate reader. Protein concentration was determined using a 50 μL aliquot of the supernatant diluted 10-fold with PBS. Relative fluorescence intensity was calculated as the ratio of total fluorescence to protein concentration. For visualization of ROS in liver tissue, frozen sections were prepared and stained using the Frozen Section ROS Detection Kit (BB-470516, Bestbio, China). Fluorescence was observed under a fluorescence microscope.

For cells: After treatment, cells were washed once with PBS and incubated with 1 mL of diluted BBoxiProbe® DHE probe at 37°C in the dark for 30 min. Cells were washed three times with serum-free medium and imaged using a confocal laser scanning microscope. Fluorescence intensity was quantified at 488/606 nm using the SpectraMax M5 reader and analyzed with ImageJ software (v1.53c, NIH, USA).

7. **MMP assay**

Mitochondria were isolated from liver tissue using the Mitochondria Extraction Kit (SM0020, Solarbio, China). Fresh liver tissue was rinsed with PBS and homogenized in lysis buffer. The homogenate was centrifuged twice at 1,000 × g for 5 min at 4°C. The supernatant was then centrifuged at 12,000 × g for 10 min at 4°C to pellet the mitochondria. After washing, the mitochondrial pellet was resuspended in storage buffer and used immediately or stored at -80°C.

Mitochondrial membrane potential was measured using the JC-1 MMP Assay Kit (C2006, Beyotime, China). A stock solution of JC-1 (200×) was diluted with ultrapure water at a ratio of 50 μL per 8 mL, followed by vigorous mixing. Then, 2 mL of JC-1 staining buffer (5×) was added and mixed thoroughly to prepare the JC-1 staining solution. This solution was further diluted 5-fold with JC-1 staining buffer (1×). A 0.1 mL aliquot of purified mitochondria (containing 10-100 μg total protein) was added to 0.9 mL of the diluted JC-1 staining solution. After mixing, fluorescence was measured using a microplate reader (Spark, Tecan, Switzerland) at excitation/emission wavelengths of 485/590 nm. Fluorescence intensity (F) per sample was recorded and normalized to total protein content (mg). The F/mg value reflects the relative proportion of mitochondrial aggregates and monomers, indicating mitochondrial membrane potential and overall health.

8. **ATP assay**

ATP levels in mouse liver tissues or HepG2 cells were measured using the Enhanced ATP Assay Kit (S0027, Beyotime, China) following the manufacturer’s protocol. Equal amounts of tissue homogenate or cell lysate from each group were used. Standard solutions were prepared at concentrations of 0.1, 0.3, 1, 3, and 10 µM. A 20 µL aliquot of each standard or sample was mixed with 100 µL of ATP detection reagent. Luminescence was measured using a microplate reader (Spark, Tecan, Switzerland). ATP levels were normalized to protein concentration, and the relative luminescence intensity (RLU/mg protein) was calculated for each sample.

9. **FAO capacity assay**

FAO capacity was measured using a Fatty Acid Oxidation Colorimetric Assay Kit (E-BC-K784-M, Elabscience, China), following the manufacturer’s instructions. All reagents were equilibrated to 25°C before use, and working solutions were prepared as directed. Cell lysates were prepared at a ratio of approximately 1 × 10⁶ cells to 200 μL of extraction buffer, homogenized, and centrifuged at 14,000 × g for 10 min at 4°C. The resulting supernatant was kept on ice and assayed within 4 h, with a portion reserved for protein quantification. The initial absorbance (A₁) of each sample was measured at 450 nm using a microplate reader. After incubation at 37°C for 30 min, the absorbance (A₂) was measured again. A standard curve was generated using A₂ values. FAO activity was calculated using the formula: FAO activity (U/g protein) = [(ΔA₄₅₀ − b) ÷ a ÷ T × 1000] ÷ Cpr × f.

10. **RT-qPCR**

Total RNA was extracted from mouse liver tissues or cells using the RNAsimple Total RNA Extraction Kit (DP419, TIANGEN, China) according to the manufacturer’s instructions. RNA concentration and purity were measured using a NanoDrop spectrophotometer (Thermo Scientific, USA). cDNA was synthesized from 1 μg of total RNA using the Evo M-MLV Reverse Transcription Premix Kit (AG11728, Accurate Biology, China). Quantitative PCR was performed using the SYBR® Green Pro Taq HS Premixed qPCR Kit (AG11701, Accurate Biology, China) on a real-time PCR system (Applied Biosystems, USA). Gene expression levels were normalized to Gapdh, and relative quantification was calculated using the 2^-ΔΔCt^ method. Primer sequences were listed in **Table S2**.

11. **Western blot**

Equal masses of mouse liver tissues or cells were lysed in RIPA lysis buffer (E-BC-R327; Elabscience, China) containing protease inhibitor PMSF (E-BC-R287; Elabscience) and phosphatase inhibitor Na3VO4 (E-BC-R250; Elabscience). Proteins were quantified using a BCA Protein Concentration Assay Kit (E-BC-K318-M, Elabscience, China). Equal-quality protein samples were separated on 12.5% SDS-PAGE gels (PG113, Epizyme, China), and separation electrophoresis of proteins was performed using a constant voltage of 90 V for 30 min, followed by a constant voltage of 120 V for 60 min. The proteins were then transferred to a polyvinylidene fluoride membrane (IPVH00010; Millipore, Billerica, MA, USA) using a constant current of 400 mA for 45-70 min. The membranes were then closed with protein free rapid blocking buffer (PS108P, Epizyme, China) at room temperature for 10 min on a shaker, and immunoblotted with primary antibodies (**Table S3**) overnight at 4°C, and then incubated with suitable HRP-coupled secondary antibodies (A21010/A21020, Abbkine, China) for 1 h at room temperature. Finally, enhanced ECL reagent (WBKLS0100, Millipore, Billerica, MA, USA) was used, and the signals were detected using FUSION FX. EDGE Imaging System (Vilber, Italy).

Cells were lysed using NP-40 buffer (P0013F, Beyotime, China) supplemented with protease and phosphatase inhibitors. Co-IP was performed using the Co-IP Kit (88804, Thermo Fisher Scientific, USA) following the manufacturer’s instructions.
